# Supplementary material for: How did communities in North West England respond to the COVID-19 lockdown? Findings from a diary study
Source: BMJ Open. 2022 Sep 19;12(9):e057774. doi: 10.1136/bmjopen-2021-057774 (PMC9485639; doi:10.1136/bmjopen-2021-057774)
Supplement: Author's manuscript [file bmjopen-2021-057774.draft_revisions.pdf]

BMJ Open is committed to open peer review. As part of this commitment we make the peer review history of every article we publish publicly available.

When an article is published we post the peer reviewers' comments and the authors' responses online. We also post the versions of the paper that were used during peer review. These are the versions that the peer review comments apply to.

The versions of the paper that follow are the versions that were submitted during the peer review process. They are not the versions of record or the final published versions. They should not be cited or distributed as the published version of this manuscript.

BMJ Open is an open access journal and the full, final, typeset and author-corrected version of record of the manuscript is available on our site with no access controls, subscription charges or pay-per-view fees (http://bmjopen.bmj.com).

If you have any questions on BMJ Open's open peer review process please email info.bmjopen@bmj.com

BMJ Open

Community responses to the UK COVID-19 lockdown: Findings from a diary study

Journal:	*BMJ Open*
Manuscript ID	bmjopen-2021-057774
Article Type:	Original research
Date Submitted by the Author:	06-Oct-2021
Complete List of Authors:	Ward, Fiona; Lancaster University, Applied Research Collaboration North West Coast<br>Halliday, Emma; Lancaster University, Applied Research Collaboration North West Coast<br>Holt, Vivien; Lancaster University, Applied Research Collaboration North West Coast<br>Khan, Koser; Lancaster University, Applied Research Collaboration North West Coast<br>Sadler, Gill; Lancaster University, Applied Research Collaboration North West Coast<br>Wheeler, Paula ; Lancaster University, Applied Research Collaboration North West Coast<br>Goldthorpe, Joanna; Lancaster University, Applied Research Collaboration North West Coast
Keywords:	COVID-19, PUBLIC HEALTH, QUALITATIVE RESEARCH

SCHOLARONE™
Manuscripts

I, the Submitting Author has the right to grant and does grant on behalf of all authors of the Work (as defined in the below author licence), an exclusive licence and/or a non-exclusive licence for contributions from authors who are: i) UK Crown employees; ii) where BMJ has agreed a CC-BY licence shall apply, and/or iii) in accordance with the terms applicable for US Federal Government officers or employees acting as part of their official duties; on a worldwide, perpetual, irrevocable, royalty-free basis to BMJ Publishing Group Ltd ("BMJ") its licensees and where the relevant Journal is co-owned by BMJ to the co-owners of the Journal, to publish the Work in this journal and any other BMJ products and to exploit all rights, as set out in our licence.

The Submitting Author accepts and understands that any supply made under these terms is made by BMJ to the Submitting Author unless you are acting as an employee on behalf of your employer or a postgraduate student of an affiliated institution which is paying any applicable article publishing charge ("APC") for Open Access articles. Where the Submitting Author wishes to make the Work available on an Open Access basis (and intends to pay the relevant APC), the terms of reuse of such Open Access shall be governed by a Creative Commons licence – details of these licences and which Creative Commons licence will apply to this Work are set out in our licence referred to above.

Other than as permitted in any relevant BMJ Author's Self Archiving Policies, I confirm this Work has not been accepted for publication elsewhere, is not being considered for publication elsewhere and does not duplicate material already published. I confirm all authors consent to publication of this Work and authorise the granting of this licence.

For peer review only

Title: Community responses to the UK COVID-19 lockdown: Findings from a diary study

Fiona Ward[1], Emma Halliday[1], Vivien Holt[1], Koser Khan[1], Gill Sadler[1], Paula Wheeler[1] Joanna Goldthorpe[1, 2]

[1]Division of Health Research, Faculty of Health & Medicine, Lancaster University, Lancaster, LA1 4YG.

[2] Corresponding author

Address for corresponding author

Division of Health Research, Faculty of Health & Medicine, Lancaster University, Lancaster, UK, LA1 4YG.

Email: j.goldthorpe@lancaster.ac.uk

For peer review only

ABSTRACT

During the COVID-19 pandemic UK communities were encouraged to help those who were vulnerable or in need. The nature, potential effects and longevity of these community responses have not been widely documented and explored. We asked 15 individuals in North West England to keep a diary during the first UK COVID-19 lockdown. Over 8 weeks, diaries were completed and supported with weekly calls with researchers. A community capacity building framework was used to explore reported community responses to the Covid pandemic. Diarists described community characteristics that enabled and hindered helpful responses in the lockdown context. Diarists frequently described informal approaches with residents acting alone or with near neighbours, although there were examples of community networks and residents recommencing formal volunteering activities. Diarists reported communities providing practical help and social support to vulnerable people. Participants perceived a greater sense of community, increased contact between residents and new networks during the period covered. The diaries provided valuable insights and the framework was a useful tool to explore the COVID-19 lockdown context. The findings indicate that organic capacity building took place, primarily via individual agency, highlighting the risk of communities being 'left behind' if there were not individuals or community networks available with resources to plug gaps in organisational support. Recommendations to sustain helpful responses to the pandemic include further consideration of ongoing community mobilisation, empowerment and community control within the capacity building framework.

Strengths and limitations of this study

- The study's diary methodology allowed for data to be collected in real time during the first Covid-19 pandemic lockdown, capturing authentic, in-the-moment insight from participants.
- The findings were organised around an established framework (Chaskin's framework of community capacity building).
- The findings reflected the experiences of an older, mostly female experience with previous experience of volunteering and/ or community service.

Objectives

Reaching and supporting people at risk, particularly those living in disadvantaged communities has been vital to containing the effects of the COVID-19 pandemic and reducing associated morbidity and mortality. Despite government and commercial organisations declaring that "we are all in it together"[1], not everyone experienced the pandemic in the same way due to the effects of national and international structural and systemic inequalities. The effects of the COVID-19 pandemic are being felt particularly severely by clinically vulnerable individuals and globally along racial, ethnic, and socio-economic divides[2-4]. These disproportionate effects highlight and exacerbate the damaging effect of health inequalities that existed before the pandemic and influenced the publication of the Marmot review[4]. This report examines the reasons behind the UK's status as having one of the highest COVID-19 mortality rates in Europe and describes how we can "build back fairer" by introducing polices that reduce health inequalities.

For peer review only - http://bmjopen.bmj.com/site/about/guidelines.xhtml

Research from previous infectious disease pandemics indicates that smaller scale social and community responses may be especially important in reaching vulnerable and marginalised populations. A rapid review of research into community responses to recent pandemics including Ebola, SARS, Middle East Respiratory Syndrome and H1N1[5] suggest that they may be crucial in engendering trust and goodwill in those who are suspicious of the motives of national governments and international organisations such as World Health Organisation. This trust and credibility is vital for delivering appropriate health messages[6], modelling behaviours, and establishing social norms that reduce viral transmission[7].

Public health leaders in the UK proposed and advocated for community responses to support vulnerable people during the pandemic. These included a "call to arms" for those with basic medical training to deliver frontline home support[8] and a framework for a structured 'whole systems' approaches to community-based initiatives in order to build community resilience [in addition to capacity building around virology, vaccinations and reducing viral transmission)[9]. These approaches would have been unlike most identified by Gilmore et al.[5] which found community engagement in high income countries (Australia, Canada, USA) usually entailed passive involvement such as targeted consultation with minority populations and very few had an equity focus or reported informal, locally driven activity.

To date, several quantitative studies have looked at volunteering during the pandemic. They have reported on the demographics of volunteers during the early lockdown period (people who were older, non-white and not living with partner), the categories of volunteering (formal, social action and community)[10] and how they were mobilised (social networks and social media groups)[11]. A survey carried out on behalf of the UK Department of Culture, Media & Sport and Office for Civil Society looked specifically at the nature of informal and formal volunteering. It found that participants volunteering informally were more likely to: keep in touch with someone who has difficulties getting out; carrying out tasks such as shopping and collecting medicine, and less likely to provide transport or escort to venues outside the home.[12] This research did not, however, explore the experiences of volunteers or others in their communities.

Gilmore et al.[5] concluded that future research should include a focus on documenting organic community engagement. In the published literature however, little attention has been given to these informal and unstructured responses to the COVID-19 pandemic carried out by citizens in their own localities. If we are to "build back better" and "fairer" it makes sense to capture, support and resource acceptable bottom-up, equitable models of community engagement that have potential to reduce health inequalities. The overarching aim of this study was to explore the experience of adults living in North West England, including perspectives on their local community's responses during the COVID-19 lockdown.

METHODS

Design

This study used solicited diaries, a research method employed in previous crisis situations to record personal experiences and observations[13, 14]. Weekly calls complemented the written diaries as these have been found to aid diary completion and improve the quality of the information recorded[15].

Setting

For peer review only - http://bmjopen.bmj.com/site/about/guidelines.xhtml

The study was undertaken by researchers from the Applied Research Collaboration North West Coast (ARC-NWC), a collaboration of universities, NHS providers and commissioners, local authorities and third sector organisations which support applied research that responds to the needs of local populations and health and care systems. Public and community involvement is central to this work and the study participants were ARC-NWC public contributors or 'Public Advisers'. The participants all lived in communities based in the North Western coastal area of England.

Participants

All Public Advisers registered with ARC-NWC were invited, by email, to take part in the study. This means that the participants and researchers may have met previously. A total of 16 people expressed an interest, although one withdrew before the diaries commenced. The remaining 15 completed the 8-week study. All were aged over 18 years. Although not purposively sampled, there was diversity in the circumstances of those who took part. They included people living alone, with families, in clinical at-risk groups, shielding households and people with caring responsibilities. Characteristics of participants are shown in Table 1.

Patient and Public Involvement

ARC NWC has paid public advisors who work alongside researchers at each stage of the research. Each ARC NWC theme (research team) has a public advisor as a co-lead to lead on prioritisation and public involvement. Teams involved in specific research studies and topics recruit additional public advisors with lived experience in relevant areas. For this study, public advisors were involved in prioritising, designing the research member checking the analysis in addition to being participants in the research.

Table 1: Characteristics of participants

For peer review only

Age	N	Employment status	N	Gender	N
30-39	*1*	Self employed	*2*	Female	*10*
40-49	*2*	Retired	*4*	Male	*3*
50-59	*5*	Not employed - carer	*2*	Not stated	*2*
60-69	*2*	Not employed - other	*5*		
70	*3*	Not stated	*2*		
Not stated	*2*				

Ethnicity		Disability	N
White – British	*9*	Yes	*5*
Black African and White	*1*	No	*6*
Pakistani	*3*	Prefer not to say	*2*
Not stated	*2*	Not stated	*2*

Procedure

A link to the diary template (created on Qualtrics online survey software) was emailed to the participants (hereafter referred to as diarists) each Monday for 8 weeks from 20th April 2020. A Microsoft Word version was also available. The diary format used a combination of closed questions which enabled diarists to rate their general and emotional health and quality of life each week and free text for them to record, as they chose, their daily activities and general reflections about their current situation. Diarists were not routinely asked specific questions about the pandemic: the exception was in week 7 when they were asked to reflect on major change in lockdown rules.

Each diarist was paired with a researcher (EH, KK, FW, PW) for an induction session and the weekly calls. The conversation during the telephone or video calls, which lasted approximately 30 minutes, was guided by a proforma which included prompts about general wellbeing and any standout events during the week. Calls were not recorded but notes taken and these were included in the analysis. At the end of the diary period, diarists took part in a focus group which explored their experience of the diary method and participating in the study. To enable diarists to speak openly, one focus group (95 minutes) was facilitated by researchers not involved in the weekly calls (VH, GS). There were 11 participants.

Analysis

The data from 115 diary-weeks, fieldnotes from 114 calls and the focus group transcript were analysed using NVIVO-12 to organise the data. (EH, VH). An inductive thematic analysis was used to organise the data according to patterns identified by the coders (FW, EH) and to draw out similarities and differences across participants' experiences[16]. This was followed by a deductive, framework analysis[17] using Chaskin's framework[18] (FW) and the use of narrative techniques to investigate diarists' experience, including their observations and personal involvement, of community responses over time. Chaskin described community capacity as:

"…the interaction of human capital, organizational resources, and social capital existing within a given community that can be leveraged to solve collective problems and improve or

For peer review only

For peer review only - http://bmjopen.bmj.com/site/about/guidelines.xhtml

maintain the well-being of a given community. It may operate through informal social processes and/or organized effort." (18, p.295).

To establish trustworthiness of the findings[19], the research team met to discuss the analysis and diarists participated in a workshop to discuss preliminary findings[20]. Quotations used here to illustrate findings are referenced using the diarist's unique ID (1-16) and the diary week (1-8). Where data comes from weekly calls, this is stated (e.g. *Diarist5-Week1-call*).

RESULTS

In reporting the findings from the Diary Project, the dimensions of the definitional framework have been reorganised to lead with the conditioning influences (Figure 1). The study took place early in the first lockdown and consequently, the context was critical.

For peer review only

Figure 1. Chaskin framework dimensions applied to Diary Project findings

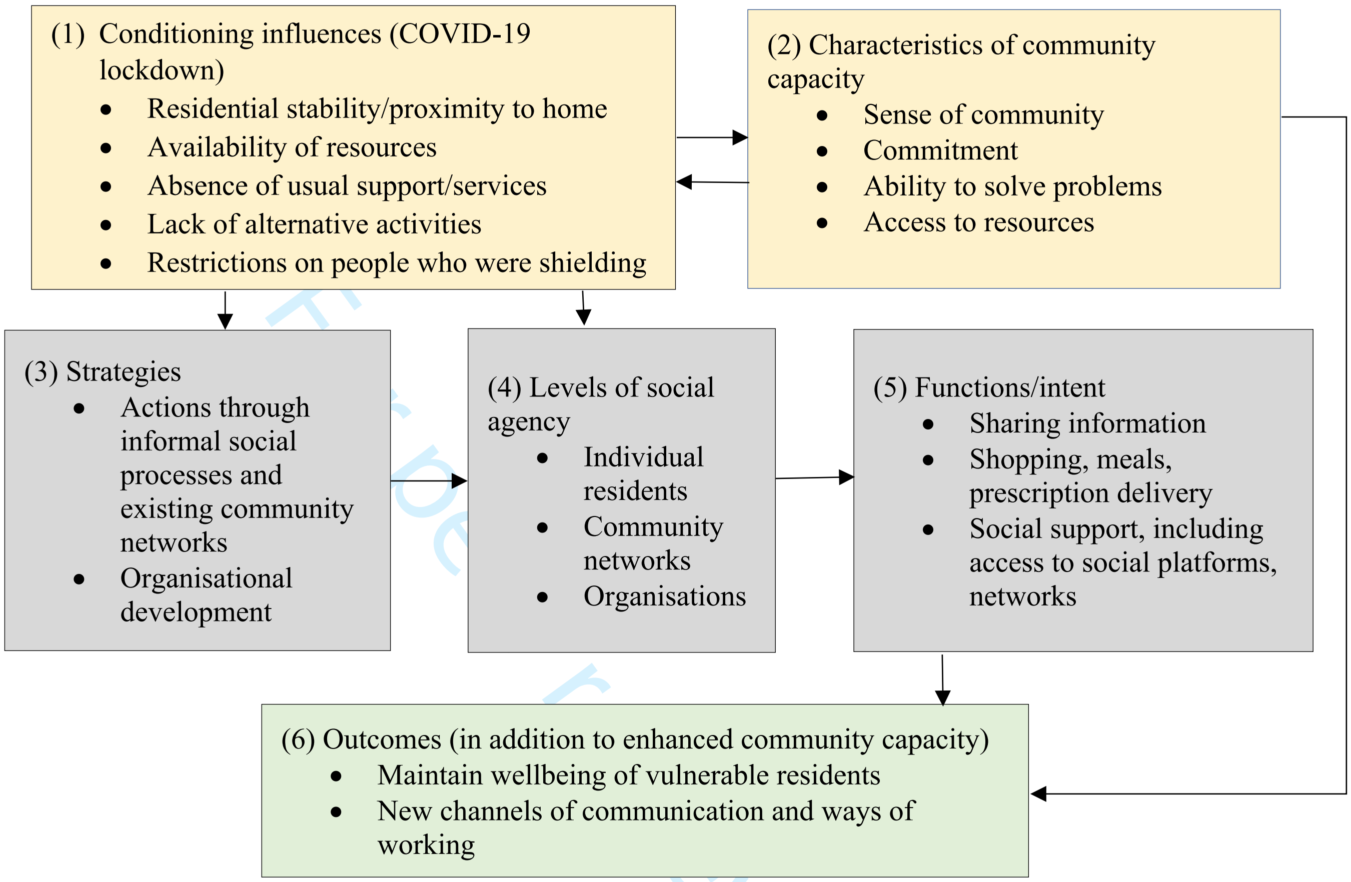

For peer review only - http://bmjopen.bmj.com/site/about/guidelines.xhtml

COVID-19 lockdown: the overriding context

Conditioning influences (dimension 1) are the *"mediating circumstances that may facilitate or inhibit community capacity and efforts to build it"* (18 p299). Although the Diary Project participants lived in different neighbourhoods, there were common factors that encouraged and inhibited the development of community capacity during this time.

Perceptions of community and facilitators of community capacity

The diarists' commentaries reflected their observation and experience of existing community conditions as well as the development of characteristics underlying community capacity, described by Chaskin as a sense of community and individuals who are committed to it, the ability to solve problems and access to resources.

The findings suggest that during the early lockdown there was, in general, a growing sense of connectedness and a recognition of mutuality of circumstances. This was often a physical thing, facilitated by residents meeting one another, but also occurred via online groups and telephone calls. One diarist, for example, reflected in a weekly call on the response when a funeral precession passed along their street saying '*it brought everyone out to pay respects as it passed. It is unusual for the place to be so busy during the day and they felt it showed the sense of community spirit that has emerged'*: they suggested that this indicated *'how much community spirit there was in the locality which people hadn't realised'* (Diarist1-week1-call).

Under national lockdown rules people were permitted to exercise daily, and this was seen as an opportunity for social connectivity, particularly for those who lived alone. Several diarists said there were more people walking locally, resulting in additional contacts and casual conversations. As one diarist wrote, 'w*hen I take my daily exercise, people are being more friendly and actually saying hello and even stopping to have a bit of chat*' (Diarist10-week1). During a catch-up call, diarists said they were *'uplifted'* by such greetings and people acknowledging one another. For some, religious occasions which could not be celebrated in the usual way also provided an opportunity for neighbours to connect: one diarist described that during the festival of Shavuot, a neighbour celebrated by sharing food with others on the street (Diarist2-week6) and similar activity was reported during Ramadan.

Several diarists wrote that more frequent conversations between neighbours had resulted in a greater level of understanding about the needs of people at risk in their communities. One diarist, for example, said that when they were delivering food parcels '*… many residents are now asking for other help/support/advice, TV licenses, Utility bill payments etc'* (Diarist11-Week2) and another described how a WhatsApp group had '*been a way to identify/support people perceived to be at risk on an informal basis, such as a group member with mental health'* (Diarist1-Week1-call). Another diarist described how their father's neighbours had responded to the crisis:

> *"My dad has always had good neighbours but now they have become fantastic...* [they] *have joined together to make sure all the old and vulnerable people are supported both emotional and practically"* (Diarist9-week4).

Changes in routine, the furlough scheme and the lack of alternative activities created human capacity and facilitated local community engagement, illustrated by one diarist who said that the suspension of their previous volunteering roles had *'led them to find other things to do to support people'* (Diarist1-Week1-call). Diarists also described the additional resources that

For peer review only - http://bmjopen.bmj.com/site/about/guidelines.xhtml

were made available to support community initiatives including donations from individuals, businesses, and social enterprises and in one case, local authority funds. One diarist involved in a community response, for example, described donations of food from local restaurants, meals being prepared in a school kitchen and resources being gathered and packaged in space made available by a community hub (Diarist11-Week1). Others referred to restaurants and cafés delivering to vulnerable people (Diarist10-Week1) and community hampers for a local nursing home (Diarist14-Week1).

Diarists demonstrated commitment through the regularity of their activities with some engaged almost daily in community responses. During the early weeks of the lockdown, organisations often suspended support services and many people, particularly those who were shielding, were isolated and struggled to get obtain essentials such as food shopping. It was clear that despite any personal difficulties' diarists were contending with, they were still thinking about maintaining contact and providing practical help to others in their community. In several cases, they supported people they had met through previous volunteer roles: one '*buddied up'* for walks in the park and another took things to someone whilst they were in hospital and checked their house.

Access to resources such as telephones and the internet facilitated the development of community capacity. Despite physical restrictions for some, diarists described telephone calls with isolated friends, family and neighbours. Another common theme was the way that diarists adapted, using forms of communication that were new to them, such as Zoom, Microsoft Teams and What's App to maintain existing connections and engage in new activities such as religious services, fundraising, individual and street level support, and volunteering activities.

Several diarists described their continued involvement with third sector organisations that they volunteered with and the sense of purpose these activities provided. Many activities had ceased because face-to-face contact was not an option but during the period covered by the diary project, some were re-configured and moved online. When the pilot call for a support group went well, one diarist described it as *'great news!'* When they also received an email asking if they were interested in helping with a new telephone support line they *'signed up straight away and awaited my first client for a phone call on Thursday'* (Diarist1-Week3).

Factors which inhibited community capacity building

At an individual level, some diarists reflected on the sense of loss they felt by not being able to undertake volunteering activities that were '*a big part of my life'* (Diarist6-Week5-call). This was particularly the case for those who were shielding. The change from their normal activities was especially difficult as one diarist wrote:

> *"Inclusion is the biggest value - we are a couple with careers based on helping people be at their best. But now can't be part of this... The sense of being part of a community and not being able to contribute when you would normally be in that role - it is frustrating - feel a bit jealous not being part of the community coming together"* (Diarist4-Week1).

At the end of the Diary Project they were still feeling a sense of loss and isolation:

> *"I love getting 'stuck into' work and doing things - nearly always for others. I am finding it exceptionally hard not to be able to do this being shielded. I had to call on a*

For peer review only - http://bmjopen.bmj.com/site/about/guidelines.xhtml

> *local volunteer to get my prescriptions from the chemist and whilst it was wonderful to receive the help it also made me more frustrated that I wasn't the one doing this!"* (Diarist4-Week8).

There was less of a sense of community for some because of where they lived. Two diarists who were also shielding described their neighbourhood as consisting of larger houses with older neighbours who rarely saw each other and another lived in a rural location, out of sight of other houses. And for some diarists, the perceptions of a mutuality of circumstances also faded as time progressed. Towards the end of the study there was frustration expressed at the response of some residents when the lockdown restrictions lifting, which arguably worked against a sense togetherness at this point.

Actors, activities, and strategies: delivering the community response

Strategies are the means through which capacity is built or engaged (dimension 3). Chaskin suggests these often include a combination of community organising, organisational and leadership development or through fostering collective relationships amongst organisations. Strategies may operate through informal social processes, organised community processes or formal targeted efforts and, to convert these strategies into action, community capacity is engaged through agency stemming from individuals, networks, and organisations (dimension 4). The 'intent' of the community capacity building is described as the function (dimension 5).

Although there was a degree of strategic development, the most frequently reported approaches to community capacity building operated through informal interactions with individual residents proactively applying knowledge, skills, and resources. Ten diarists described the instrumental and emotional support they provided (or observed others providing), often to those who were elderly or shielding. Throughout the eight-week study, there were frequent descriptions of *'people helping each other out'* (Diarist1-Week3-call). Activities or functions included practical tasks (e.g. shopping, fetching prescriptions and delivery medicines) and providing support through socially distanced walks and telephone calls.

Community capacity was also developed through networks of relationships with neighbourhood groups and organisations acting together. Several diarists described their membership of social media groups which provided a forum for requests and offers of help from one resident to another. One diarist described how they identified a need for this and set up a street-level WhatsApp group, saying they were *'enjoying being able to support my street through social media. There is always something to offer from shopping to advice'* (Diarist 14-Week1). Another diarist was heavily involved in a response co-ordinated by a town council. This approach was enabled by local government funding and individuals who were already active in their community mobilised social enterprises, businesses, and other local service providers to provide essential shopping and meals to residents at risk. Their local knowledge, ability to act quickly and being known in the community were seen to be essential components underlying their rapid community response (Diarist11-Week1-call).

At an organisational level, Chaskin suggested that the development of community capacity *"might be reflected in the ability of such organizations to carry out their functions responsively, effectively, and efficiently as part of the larger system of actors and processes to which they are connected, within and beyond the community"* (18 p.298). Positive reporting of organisational community capacity building in the diaries largely focused on

For peer review only - http://bmjopen.bmj.com/site/about/guidelines.xhtml

organisations adapting support service to be delivered online and there was enthusiasm when, a few weeks after the commencement of lockdown, diarists were able to engage in volunteering roles. This included one diarist who became very actively involved in an initiative to support people with mental health problems and a befriending project. There was also sympathy expressed for the response of professionals such as general practitioners and the clergy who were seen to be *'reaching out a much as they can but they can't fill the gaps'* (Diarist4-Week8).

But during the initial stages of lockdown, several diarists expressed the view that the needs of vulnerable residents were most frequently being met by individuals or community networks. Diarists expressed concern that organisations needed to find alternative ways to provide for service users reliant on them for social contact and support (e.g. Diarist1-Week2-call) and several suggested that organisations had often been slow to respond to the needs of the community or provide guidance to inform community-based action. Frustration was expressed about what was seen as the lack of effective response from *'well paid people'*: one diarist described this as *'a disconnect between 'grassroots' organisations and individuals doing stuff, and… councils and others talking about the doing'* (Diarist11-Week1-call) and another wrote about a lack of co-ordination when they did react:

> *"…there are numerous voluntary organisations that do not like working with each other, they seem to have lost focus as to why they exist. They all share one thing in common, they have definitely forgotten that they exist to serve and support the community and if an individual organisation can't provide that support, then signpost them to one which can"* (Diarist10-Week2).

It was also suggested that in some cases, organisations were unable to utilise potential community action because of pre-existing organisational arrangements, thus inhibiting the potential impact of local groups. One diarist reported that statutory funding was not available to community groups who were ready to respond to local need because they were not formally constituted. This was at a time when some organisations which could have been part of the community network had furloughed staff, so restricting the availability of people in key roles.

Outcomes of the increased community capacity

The outcome of the community activity (dimension 6) as identified and described by the diarists included an increase in community capacity (dimension 2) as well as outcomes relating to the tasks undertaken: primarily these were maintaining the wellbeing of residents at risk. Clearly this support was valued as one diarist wrote:

> *"People are mentioning that they will really miss us tomorrow!!! It's a very humbling feeling, knowing that a small meal, a chat and a smile can mean so much to someone…"* (Diarist11-Week3)

The overall perception from the diarists was that there was a greater sense of community, increased contact between residents and that the development of new networks and channels of communication had resulted in a greater awareness of the needs of individuals within the community, information sharing and pathways for individuals and businesses to '*help out more'*. Diarists also described, through their own volunteering experiences, the new initiatives and ways of working developed by the organisations they were connected with to meet the support needs of their service users.

For peer review only - http://bmjopen.bmj.com/site/about/guidelines.xhtml

Several diarists described the importance to their personal wellbeing of being engaged in community or volunteering work at such a challenging time. They described these activities as *'giving a sense of purpose'* (Diary3 wk2) and enhancing feeling of self-worth through being *'useful' 'of value'* and *'productive'* (Diarist11-Week1). Another diarist described the mutual benefit of being involved in a new support initiative:

> *"Had my first Check in and chat call today, went brilliantly on the phone for an hour and I think we both got something out of the experience. Look forward to my next chat next week"* (Diarist1-Week3).

But this activity also had personal physical and mental consequences. One diarist was heavily involved in the mobilisation of community network which collected, processed, and delivered food to vulnerable residents described how demanding it could be:

> "*Over at* [locality2] *primary school to collect pasta/soups to package and distribute later today. 11.00am, 120 food/sandwiches packages ready to load onto van, the list is growing, some residents ask to be removed as they have adequate support networks, but for every 1 we take off, 4 more ask for support. Finished delivering food around 5.45pm, really tired*" (Diarist11-Week2).

In addition to being active and engaged in proving support to others, diarists were also experiencing the pandemic and were dealing with the psychological impact of this. This diarist also noted the additional strain associated with being out in the community and having contact with vulnerable people:

> *"Just been told that today, will be our final day of delivering support as part of the* [organisation] *COVID-19 community response. Such a mixture of feelings. Relieved, and happy that I (along with 10 of my friends and colleagues) have so far survived contracting the virus, despite placing ourselves (and our loved ones) in danger. The stress of being in close proximity with vulnerable residents, many of them elderly, with multiple and complex health conditions has been enormous at times"* (Diary11-Week7).

The nature of the Diary Study, in gathering the perceptions of 15 individuals during the early weeks of the lockdown, meant it was not possible to comment on the extent to which the community capacity built or other outcomes were sustained beyond the diary period. Even during the period covered by the Diary Study, activities were changing and in one instance, a community response that had regularly delivered shopping and meals to more than 100 people was being '*wound down'*. In part this was an apparent reaction to fears that they were *'creating dependency'* but also with the understanding that furloughed staff were returning to work and there was some return to normality.

But several diarists, as illustrated below, did express their hope that the greater sense of community and capacity built would be sustained:

> *"Making sure we don't lose the positives There have been so many positive things that have come from the Covid situation. So much connectivity between people, huge numbers offering help, old friendships renewed, new friends being made, lots of creative solutions being identified to new and difficult problems. I really hope a lot of*

For peer review only - http://bmjopen.bmj.com/site/about/guidelines.xhtml

these really beneficial things can be captured and encouraged to still remain in place when life gets a bit more back to normal" (Diarist4-Week8).

There was a hope that this enthusiasm could be captured but clearly also some doubt – with reference to the community connectedness they had observed, this diarist said '*What a shame we can't bottle it!'* (Diary4-Week3).

CONCLUSIONS

The research reported here aimed to explore community responses to the COVID-19 pandemic. Whilst the data from this Diary Study focused on emerging community responses rather than planned initiatives, Chaskin's exploration of community capacity proved to be a useful framework to understand the observations and experiences of the diarists during the early weeks of the first UK COVID-19 lockdown. Though actions were largely reactive, the findings did indicate that capacity building took place: participants described a sense of community, and a desire to be actively engaged; the generation of ideas and solutions to local issues as they were identified and harnessing of resources were apparent in data from diary entries and weekly calls.

Using Chaskin's framework also highlights the importance of conditioning influences and in the predicament created by the COVID-19 lockdown, the accounts of the diarists elucidating the critical role of context in the development of community capacity. Inhibiting influences include the lockdown rules, restrictions on people who were shielding and the absence of existing services and other sources of support. The diarists described how community capacity building was supported by people's physical proximity to home, the use of information technology, greater availability, and by goodwill which frequently appeared to engender a feeling of being 'in it together'.

The picture of the community response presented by the diaries contains insights into different population characteristics that could inform the recovery. It supports research which suggests that residents must be engaged if more systemic recovery support is to be valued e.g.(21) and reveals valuable information from members of the community about the knowledge and processes that resulted in the provision of essential support whilst there were gaps in service delivery. As the National Institute for Health and Care Excellence has indicated, the recovery period provides an opportunity for local government and health and care services to build on the strengths of communities to ensure initiatives are 'relevant and meaningful'[22].

The main functions of the community response described by the diarists was to provide essential resources to residents at risk, and to a lesser extent, social support. The sharing of intelligence was also required to enable this to happen, a finding in line with other work which described the *'creative solutions'* developed by residents who understand their community[23] Whilst diarists wrote about the communication of lockdown rules and government health messages, there was no evidence that this formed any part of their activities, contrasting with the type of activities described in previous epidemics[7]. This finding supports the UK Government's research that found informal volunteers were most likely to be employed maintaining social contact with less mobile neighbours and carrying out practical tasks such as shopping and collecting medicine[12].

Support was provided through community organising, primarily via individual agency and local connections and, to a lesser extent, organised community processes. This resonates with

For peer review only - http://bmjopen.bmj.com/site/about/guidelines.xhtml

other research findings[5] where the main actors in community pandemic responses were community leaders, community and faith-based organisations, community networks, committees, and individuals. Some diarists referred to targeted efforts of larger organisations but in general the activities described were not part of a strategic approach, rather they were informal and evolving in response to the initial crisis and rapidly changing circumstances. The prominence of these individual and self-organised street-level activities appeared to be effective for those residents who were identified: practical support was provided at a time when formal support services were not available or not able to cope. Further supporting the findings of Gilmore et al.[5] there was no discussion in the diaries about community involvement in wider strategic decision-making.

The greater community capacity generated during the time covered by the Diary Study appears to have been created through a wider awareness of need within the community and the development of channels to share this information and respond to need (e.g. through social media groups). Other outcomes include access to new sources of support such as co-walking and befriending which enabled the wellbeing of residents at risk to be maintained. It is also important to acknowledge the personal toll for those involved in a community response: volunteers were similarly experiencing the lockdown with its associated strain on themselves, their families, and acquaintances in addition to experiencing anxieties about their contact with infected and clinically vulnerable people. This raises questions about appropriate support and the resources required to sustain successful elements of community-based responses during the COVID-19 recovery and beyond.

In contrast with other community action frameworks, the concepts of empowerment and control do not feature prominently in Chaskin's dimensions of community capacity building. Similarly, the processes and outcomes described by the diarists did not include references to feelings of empowerment and control. One apparent reason for this may be that the unique context generated by the COVID-19 lockdown meant that the singular concern of many individuals, networks and organisations was to meet the immediate needs of residents most at risk. Consequently, in this context, Chaskin's framework was useful to describe this *'inward gaze'*[24] with its focus on, for example, community capacities, and neighbourhood conditions. But moving forward, and beyond the COVID-19 recovery, it is argued that power and control need to be integral elements in the consideration of community capacity, requiring an *'outward gaze'* on the political and social change required to enable structural gaps and inequalities to be addressed.

The risk of community capacity building being considered out with empowerment and control is a particular concern at a time when there are reductions in state spending and, as Popay et al.[24] argue, pressure to focus on *'equipping communities to use their 'assets' to manage 'shocks' like COVID-19—to adapt to, rather than transform, existing inequalities'* (p.8). With reduced public expenditure, process of 'citizen-shift' has also been described whereby interventions are increasingly individualised and service providers are enabled to create upstream energy from grassroots level to mobilise against inequalities[25]. This is an important area for future research and could inform strategies to "build back better".

Some potential limitations of the study should be noted. Firstly, the small participant sample (n=15), consisted of an older age group and their roles as Public Advisers means that findings should be considered in the context of this study. Though they were from a range of backgrounds and with differing personal circumstances, they were all previously connected with volunteering and community networks in various ways. Second, although the findings of

For peer review only - http://bmjopen.bmj.com/site/about/guidelines.xhtml

this study suggest that community capacity building took place, the short-term nature of the study means it is not possible to assess the extent to which it was sustained or whether there was a lasting impact. Finally, the diary entries and calls also do not provide a complete picture of activity with local communities, rather they are the observations and experiences these individuals chose to report: the method adopted meant the agenda was set by diarists and researchers did not ask diarists about any possible 'gaps' in their accounts.

There have been few qualitative diary studies completed during crisis situations, and the findings of this research suggest that the combination of written diaries and weekly calls was a useful way to gather the experiences and observations of participants during the COVID-19 lockdown. The study also contributes to building wider knowledge around community responses in the COVID-19 pandemic.

The findings illuminate the community responses experienced and observed at a time of considerable uncertainty and threats to wellbeing resulting from the COVID-19 pandemic. Chaskin's community capacity building framework focused analytical attention on the conditional influences and underlying characteristics of community capacity in addition to the strategies, actors, intent, and outcomes. This demonstrated the complexity of the situation and the central importance of the specific context and of individual agency: residents were engaged (sometimes intensively) in developing links, solutions, working with what they had and, in some cases, with organisations that were in a position to respond. This confirms the likelihood that many communities could be left behind if there were not equipped individuals and/or community networks able fill the gaps in the organisational response, at least in the short-term. This could lead to increased health inequalities.

A number of directions for future research have emerged from the study. These include insights from residents who began their volunteering journey during the pandemic and looking at the impact of the community response on the wellbeing of those involved as well as those being supported. Investigating whether individuals and community networks involved in the immediate response were mobilised in the recovery post pandemic would be valuable: embedding an *'outward gaze'* into future research could also ascertain the extent to which community empowerment and involvement in wider decision-making results in strategic dimension to community capacity building post COVID-19. Identifying commonalities or differences in experiences across communities could also provide valuable information about inhibiting influences for 'communities left behind'.

FUNDING
This research was funded by the National Institute for Health Research (NIHR) Applied Research Collaboration North West Coast (ARC-NWC). The views expressed are those of the author(s) and not necessarily those of the NHS, the NIHR or the Department of Health and Social Care.

ETHICS APPROVAL
Ethics approval was granted by Lancaster University Faculty of Health and Medicine's Ethics Committee in April 2020 (FHMREC19076).

CONFLICT OF INTERESTS
The author(s) declared no potential conflicts of interest with respect to the research, authorship and/or publication of this article.

For peer review only - http://bmjopen.bmj.com/site/about/guidelines.xhtml

AUTHOR CONTRIBUTION

Fiona Ward designed the study, developed the protocol, co-ordinated the data collection, analysed the data, wrote the manuscript; Emma Halliday designed the study, collected and analysed the data, reviewed the manuscript. Vivien Holt collected the data and reviewed the manuscript; Koser Khan collected the data and reviewed the manuscript; Gill Sadler collected the data; Paula Wheeler collected the data and reviewed the manuscript; Joanna Goldthorpe wrote the manuscript and provided methodological and analytical support.

The authors wish to thank all ARC-NWC Public Advisers who participated in the study and to Alan Price and Neil Joseph who reviewed the paper. Thanks also to Olga Angulo-Judez, Lancaster University, who provided technical support and training for diarists in the use of online methods.
This paper is dedicated to the memory of Gill Sadler who sadly passed away in January 2021.

DATA AVIAILABILITY STATEMENT

The data underlying this article cannot be shared publicly due to the need to protect the privacy and confidentiality of individuals that participated in the study. The data will be shared on reasonable request to the corresponding author.

REFERENCES

1. Sobande F. 'We're all in this together': Commodified notions of connection, care and community in brand responses to COVID-19. *European Journal of Cultural Studies*. 2020;23(6):1033-7.
2. Bambra C, Riordan R, Ford J, et al. The COVID-19 pandemic and health inequalities. *J Epidemiol Community Health*. 2020;74(11):964-8.
3. Mishra V, Seyedzenouzi G, Almohtadi A, et al. Health inequalities during COVID-19 and their effects on morbidity and mortality. *Journal of healthcare leadership*. 2021;13:19.
4. Marmot M, Allen J, Goldblatt P, et al. Build back fairer: the COVID-19 Marmot review. The pandemic, socioeconomic and health inequalities in England. 2020.
5. Gilmore B, Ndejjo R, Tchetchia A, et al. Community engagement for COVID-19 prevention and control: a rapid evidence synthesis. *BMJ global health*. 2020;5(10):e003188.
6. Hocevar KP, Metzger, M., & Flanagin, A. J. Source Credibility, Expertise, and Trust in Health and Risk Messaging. Oxford Research Encyclopedia of Communication: Oxford University Press; 2017.
7. Ghio D, Lawes-Wickwar S, Tang MY, et al. What influences people's responses to public health messages for managing risks and preventing infectious diseases? A rapid systematic review of the evidence and recommendations. 2020.
8. Haines A, de Barros EF, Berlin A, et al. National UK programme of community health workers for COVID-19 response. *The Lancet*. 2020;395(10231):1173-5.
9. South J, Stansfield J, Amlôt R, et al. Sustaining and strengthening community resilience throughout the COVID-19 pandemic and beyond. *Perspectives in Public Health*. 2020;140(6):305-8.
10. Mak HW, Fancourt D. Predictors of engaging in voluntary work during the Covid-19 pandemic: analyses of data from 31,890 adults in the UK. *Perspectives in Public Health*. 2020:1757913921994146.
11. Carlsen HB, Toubøl J, Brincker B. On solidarity and volunteering during the COVID-19 crisis in Denmark: the impact of social networks and social media groups on the distribution of support. *European Societies*. 2021;23(sup1):S122-S40.

For peer review only - http://bmjopen.bmj.com/site/about/guidelines.xhtml

12. Department of Digital C, Media & Sport & Office for Civil Society. A Look at Volunteering during the Response to COVID-19. 2021. A look at volunteering during the response to COVID-19 2021.
13. Medd W, Deeming H, Walker G, et al. The flood recovery gap: a real-time study of local recovery following the floods of June 2007 in Hull, North East England. *Journal of Flood Risk Management*. 2015;8(4):315-28.
14. Mort M, Convery I, Baxter J, et al. Psychosocial effects of the 2001 UK foot and mouth disease epidemic in a rural population: qualitative diary based study. *BMJ*. 2005;331(7527):1234.
15. Herron R, Dansereau L, Wrathall M, et al. Using a flexible diary method rigorously and sensitively with family carers. *Qualitative health research*. 2019;29(7):1004-15.
16. Braun V, Clarke V. Using thematic analysis in psychology. *Qualitative research in psychology*. 2006;3(2):77-101.
17. Srivastava A, Thomson SB. Framework analysis: a qualitative methodology for applied policy research. 2009.
18. Chaskin RJ. Building community capacity: A definitional framework and case studies from a comprehensive community initiative. *Urban affairs review*. 2001;36(3):291-323.
19. Peters S. Qualitative research methods in mental health. Royal College of Psychiatrists; 2010.
20. Birt L, Scott S, Cavers D, et al. Member checking: a tool to enhance trustworthiness or merely a nod to validation? *Qualitative health research*. 2016;26(13):1802-11.
21. Harkins C. Supporting community recovery and resilience in response to the COVID-19 pandemic–a rapid review of evidence. Glasgow; 2020.
22. Maiden H, Jagroo J, Shearn P, et al. Spotlight on community engagement: NICE resources in the context of COVID-19—NICE public health guidance update. *Journal of Public Health* (Oxford, England). 2021.
23. McMillan R. Learning from nine months of crisis response: Local Trust; 2020 [Available from: https://localtrust.org.uk/news-and-stories/blog/learning-from-nine-months-of-crisis-response/
24. Popay J, Whitehead M, Ponsford R, et al. Power, control, communities and health inequalities I: theories, concepts and analytical frameworks. *Health Promotion International*. 2020.
25. Williams O, Fullagar S. Lifestyle drift and the phenomenon of ‘citizen shift’ in contemporary UK health policy. *Sociology of Health and Illness*. 2019;41(1):20-35.

For peer review only

For peer review only - http://bmjopen.bmj.com/site/about/guidelines.xhtml

Table 1: Characteristics of participants

**Age**	***N***	**Employment status**	***N***	**Gender**	***N***
30-39	*1*	Self employed	*2*	Female	*10*
40-49	*2*	Retired	*4*	Male	*3*
50-59	*5*	Not employed - carer	*2*	Not stated	*2*
60-69	*2*	Not employed - other	*5*		
70	*3*	Not stated	*2*		
Not stated	*2*				

**Ethnicity**		**Disability**	***N***
White – British	*9*	Yes	*5*
Black African and White	*1*	No	*6*
Pakistani	*3*	Prefer not to say	*2*
Not stated	*2*	Not stated	*2*

For peer review only

For peer review only

Figure 1. Chaskin framework dimensions applied to Diary Project findings

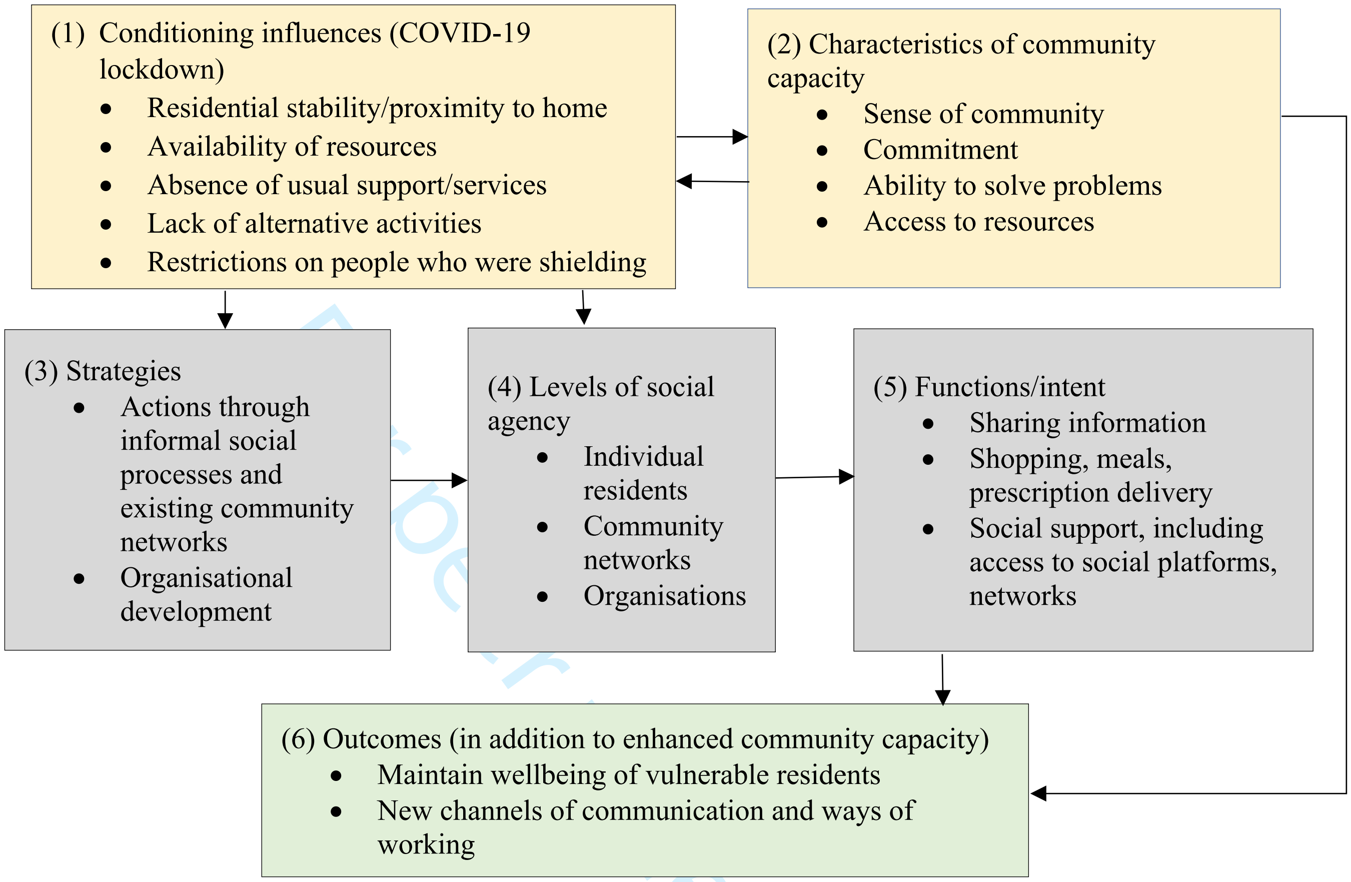

For peer review only - http://bmjopen.bmj.com/site/about/guidelines.xhtml

COREQ (COnsolidated criteria for REporting Qualitative research) Checklist

A checklist of items that should be included in reports of qualitative research. You must report the page number in your manuscript where you consider each of the items listed in this checklist. If you have not included this information, either revise your manuscript accordingly before submitting or note N/A.

Topic	Item No.	Guide Questions/Description	Reported on Page No.
**Domain 1: Research team and reflexivity**			
Personal characteristics			
Interviewer/facilitator	1	Which author/s conducted the interview or focus group?	
Credentials	2	What were the researcher's credentials? E.g. PhD, MD	
Occupation	3	What was their occupation at the time of the study?	
Gender	4	Was the researcher male or female?	
Experience and training	5	What experience or training did the researcher have?	
Relationship with participants			
Relationship established	6	Was a relationship established prior to study commencement?	
Participant knowledge of the interviewer	7	What did the participants know about the researcher? e.g. personal goals, reasons for doing the research	
Interviewer characteristics	8	What characteristics were reported about the inter viewer/facilitator? e.g. Bias, assumptions, reasons and interests in the research topic	
**Domain 2: Study design**			
Theoretical framework			
Methodological orientation and Theory	9	What methodological orientation was stated to underpin the study? e.g. grounded theory, discourse analysis, ethnography, phenomenology, content analysis	
Participant selection			
Sampling	10	How were participants selected? e.g. purposive, convenience, consecutive, snowball	
Method of approach	11	How were participants approached? e.g. face-to-face, telephone, mail, email	
Sample size	12	How many participants were in the study?	
Non-participation	13	How many people refused to participate or dropped out? Reasons?	
Setting			
Setting of data collection	14	Where was the data collected? e.g. home, clinic, workplace	
Presence of non-participants	15	Was anyone else present besides the participants and researchers?	
Description of sample	16	What are the important characteristics of the sample? e.g. demographic data, date	
Data collection			
Interview guide	17	Were questions, prompts, guides provided by the authors? Was it pilot tested?	
Repeat interviews	18	Were repeat inter views carried out? If yes, how many?	
Audio/visual recording	19	Did the research use audio or visual recording to collect the data?	
Field notes	20	Were field notes made during and/or after the inter view or focus group?	
Duration	21	What was the duration of the inter views or focus group?	
Data saturation	22	Was data saturation discussed?	
Transcripts returned	23	Were transcripts returned to participants for comment and/or	

For peer review only

Topic	Item No.	Guide Questions/Description	Reported on Page No.
		correction?	
**Domain 3: analysis and findings**			
Data analysis			
Number of data coders	24	How many data coders coded the data?	
Description of the coding tree	25	Did authors provide a description of the coding tree?	
Derivation of themes	26	Were themes identified in advance or derived from the data?	
Software	27	What software, if applicable, was used to manage the data?	
Participant checking	28	Did participants provide feedback on the findings?	
Reporting			
Quotations presented	29	Were participant quotations presented to illustrate the themes/findings? Was each quotation identified? e.g. participant number	
Data and findings consistent	30	Was there consistency between the data presented and the findings?	
Clarity of major themes	31	Were major themes clearly presented in the findings?	
Clarity of minor themes	32	Is there a description of diverse cases or discussion of minor themes?	

Developed from: Tong A, Sainsbury P, Craig J. Consolidated criteria for reporting qualitative research (COREQ): a 32-item checklist for interviews and focus groups. *International Journal for Quality in Health Care*. 2007. Volume 19, Number 6: pp. 349 – 357

Once you have completed this checklist, please save a copy and upload it as part of your submission. DO NOT include this checklist as part of the main manuscript document. It must be uploaded as a separate file.

For peer review only

Community responses to the UK COVID-19 lockdown: Findings from a diary study

Journal:	*BMJ Open*
Manuscript ID	bmjopen-2021-057774.R1
Article Type:	Original research
Date Submitted by the Author:	02-Mar-2022
Complete List of Authors:	Ward, Fiona; Lancaster University, Applied Research Collaboration North West Coast<br>Halliday, Emma; Lancaster University, Applied Research Collaboration North West Coast<br>Holt, Vivien; Lancaster University, Applied Research Collaboration North West Coast<br>Khan, Koser; Lancaster University, Applied Research Collaboration North West Coast<br>Sadler, Gill; Lancaster University, Applied Research Collaboration North West Coast<br>Wheeler, Paula ; Lancaster University, Applied Research Collaboration North West Coast<br>Goldthorpe, Joanna; Lancaster University, Applied Research Collaboration North West Coast
**Primary Subject Heading**:	Public health
Secondary Subject Heading:	Qualitative research, Health policy, Research methods, Mental health
Keywords:	COVID-19, PUBLIC HEALTH, QUALITATIVE RESEARCH

I, the Submitting Author has the right to grant and does grant on behalf of all authors of the Work (as defined in the below author licence), an exclusive licence and/or a non-exclusive licence for contributions from authors who are: i) UK Crown employees; ii) where BMJ has agreed a CC-BY licence shall apply, and/or iii) in accordance with the terms applicable for US Federal Government officers or employees acting as part of their official duties; on a worldwide, perpetual, irrevocable, royalty-free basis to BMJ Publishing Group Ltd ("BMJ") its licensees and where the relevant Journal is co-owned by BMJ to the co-owners of the Journal, to publish the Work in this journal and any other BMJ products and to exploit all rights, as set out in our licence.

The Submitting Author accepts and understands that any supply made under these terms is made by BMJ to the Submitting Author unless you are acting as an employee on behalf of your employer or a postgraduate student of an affiliated institution which is paying any applicable article publishing charge ("APC") for Open Access articles. Where the Submitting Author wishes to make the Work available on an Open Access basis (and intends to pay the relevant APC), the terms of reuse of such Open Access shall be governed by a Creative Commons licence – details of these licences and which Creative Commons licence will apply to this Work are set out in our licence referred to above.

Other than as permitted in any relevant BMJ Author's Self Archiving Policies, I confirm this Work has not been accepted for publication elsewhere, is not being considered for publication elsewhere and does not duplicate material already published. I confirm all authors consent to publication of this Work and authorise the granting of this licence.

For peer review only

Title: Community responses to the UK COVID-19 lockdown: Findings from a diary study

Fiona Ward[1], Emma Halliday[1], Vivien Holt[1], Koser Khan[1], Gill Sadler[1], Paula Wheeler[1]
Joanna Goldthorpe[1, 2]

[1]Division of Health Research, Faculty of Health & Medicine, Lancaster University, Lancaster, LA1 4YG.

[2] Corresponding author

Address for corresponding author

Division of Health Research, Faculty of Health & Medicine, Lancaster University, Lancaster, UK, LA1 4YG.

Email: j.goldthorpe@lancaster.ac.uk

For peer review only

ABSTRACT

OBJECTIVES: During the COVID-19 pandemic the UK government and public health leaders advocated for community level responses to support vulnerable people. This activity could be planned and co-ordinated, however much was informal and developed organically. The effects on the individuals who were involved in providing and receiving informal support and implications for their communities has not been widely explored. The aim of this study was therefore to document and explore the nature, potential effects and longevity of community responses to the COVID-19 pandemic. PARTICIPANTS: We asked 15 individuals in North West England to keep a diary during the first UK COVID-19 lockdown. Over 8 weeks, diaries were completed and supported with weekly calls with researchers. A community capacity building framework was used to explore reported community responses to the Covid pandemic. RESULTS: Diarists described community characteristics that enabled and hindered helpful responses in the lockdown context. Diarists frequently described informal approaches with residents acting alone or with near neighbours, although there were examples of community networks and residents recommencing formal volunteering activities. Diarists reported communities providing practical help and social support to vulnerable people. Participants perceived a greater sense of community, increased contact between residents and new networks during the period covered. CONCLUSION: The diaries provided valuable insights and the framework was a useful tool to explore the COVID-19 lockdown context. The findings indicate that organic capacity building took place, primarily via individual agency, highlighting the risk of communities being 'left behind' if there were not individuals or community networks available with resources to plug gaps in organisational support. Recommendations to sustain helpful responses to the pandemic include further consideration of ongoing community mobilisation, empowerment and community control within the capacity building framework.

Strengths and limitations of this study

- The study's diary methodology allowed for data to be collected in real time during the first Covid-19 pandemic lockdown, capturing authentic, in-the-moment insight from participants.
- The findings were organised around an established framework (Chaskin's framework of community capacity building).
- The findings reflected the experiences of an older, mostly female experience with previous experience of volunteering and/ or community service.

Objectives

Reaching and supporting people at risk, particularly those living in disadvantaged communities has been vital to containing the effects of the COVID-19 pandemic and reducing associated morbidity and mortality. Despite government and commercial organisations declaring that "we are all in it together"(1), not everyone experienced the pandemic in the same way due to the effects of national and international structural and systemic inequalities. The effects of the COVID-19 pandemic are being felt particularly

For peer review only - http://bmjopen.bmj.com/site/about/guidelines.xhtml

severely by clinically vulnerable individuals and globally along racial, ethnic, and socio-economic divides(2-4). These disproportionate effects highlight and exacerbate the damaging effect of health inequalities that existed before the pandemic and influenced the publication of the Marmot review(4). This report examines the reasons behind the UK's status as having one of the highest COVID-19 mortality rates in Europe and describes how we can "build back fairer" by introducing polices that reduce health inequalities.

Research from previous infectious disease pandemics indicates that smaller scale social and community responses may be especially important in reaching vulnerable and marginalised populations. A rapid review of research into community responses to recent pandemics including Ebola, SARS, Middle East Respiratory Syndrome and H1N1(5) suggest that they may be crucial in engendering trust and goodwill in those who are suspicious of the motives of national governments and international organisations such as World Health Organisation. This trust and credibility is vital for delivering appropriate health messages(6), modelling behaviours, and establishing social norms that reduce viral transmission(7).

Public health leaders in the UK proposed and advocated for community responses to support vulnerable people during the pandemic. These included a "call to arms" for those with basic medical training to deliver frontline home support(8) and a framework for a structured 'whole systems' approaches to community-based initiatives in order to build community resilience [in addition to capacity building around virology, vaccinations and reducing viral transmission)(9). These approaches would have been unlike most identified by Gilmore et al.(5) which found community engagement in high income countries (Australia, Canada, USA) usually entailed passive involvement such as targeted consultation with minority populations and very few had an equity focus or reported informal, locally driven activity.

To date, several quantitative studies have looked at volunteering during the pandemic. They have reported on the demographics of volunteers during the early lockdown period (people who were older, non-white and not living with partner), the categories of volunteering (formal, social action and community)(10) and how they were mobilised (social networks and social media groups)(11). A survey carried out on behalf of the UK Department of Culture, Media & Sport and Office for Civil Society looked specifically at the nature of informal and formal volunteering. It found that participants volunteering informally were more likely to: keep in touch with someone who has difficulties getting out; carrying out tasks such as shopping and collecting medicine, and less likely to provide transport or escort to venues outside the home.(12) This research did not, however, explore the experiences of volunteers or others in their communities.

Gilmore et al.(5) concluded that little attention has been given to informal and unstructured responses to the COVID-19 pandemic carried out by citizens in their own localities and thatfuture pandemic research should include a focus on documenting organic community engagement This research should additionally be of interest to readers concerned with community empowerment and place-based approaches; citizen-led community initiatives have potential to positively disrupt the power relationships inherent in top-down community development initiatives, resulting in potential benefits for public health(13). If we are to "build back better" and "fairer" it makes sense to capture, support and resource acceptable bottom-up, equitable models of community engagement that have potential to reduce health inequalities.

For peer review only - http://bmjopen.bmj.com/site/about/guidelines.xhtml

Chaskin's (2001) definition and framework for community capacity offers a useful lens through which to explore organic community responses to the recent pandemic. It provides a structure for exploration around how local resources were informally mobilised to solve neighbourhood-level problems, and the parameters of these efforts. There may be a point at which individuals and resources come together to build capacity in communities, but also a point at which this capacity is reached and more formal, planned approaches may need to be mobilised. Chaskin (2001)conceptualises community capacity as (14)
"…the interaction of human capital, organizational resources, and social capital existing within a given community that can be leveraged to solve collective problems and improve or maintain the well-being of a given community. It may operate through informal social processes and/or organized effort." (18, p.295).

The study was exploratory (meaning we did not formulate precise research questions in advance) but sought to broadly understand public experiences of the pandemic including its effects on diarists' everyday lives. This paper uses Chaskin's Framework to present perspectives on one of the prominent themes - that of local community responses to the first COVID-19 lockdown,

METHODS

Design

This study used solicited diaries, a research method employed in previous crisis situations to record personal experiences and observations(15, 16). Weekly calls complemented the written diaries as these have been found to aid diary completion and improve the quality of the information recorded(17).

Setting

The study was undertaken by researchers from the Applied Research Collaboration North West Coast (ARC-NWC), a collaboration of universities, NHS providers and commissioners, local authorities and third sector organisations which support applied research that responds to the needs of local populations and health and care systems. Public and community involvement is central to this work and the study participants were ARC-NWC public contributors or 'Public Advisers'. The participants all lived in communities based in the North Western coastal area of England.

Participants

All Public Advisers registered with ARC-NWC were invited, by email, to take part in the study. This means that the participants and researchers may have met previously. A total of 16 people expressed an interest, although one withdrew before the diaries commenced. The remaining 15 completed the 8-week study. All were aged over 18 years. Although not purposively sampled, there was diversity in the circumstances of those who took part. They included people living alone, with families, in clinical at-risk groups, shielding households and people with caring responsibilities. Characteristics of participants are shown in Table 1. Written consent was obtained from diarists, who were also reminded of their rights during each weekly call.

Patient and Public Involvement

ARC NWC has paid public advisers who work alongside researchers at each stage of the research. Each ARC NWC theme (research team) has a public adviser as a co-lead to lead on

For peer review only

prioritisation and public involvement. Teams involved in specific research studies recruit additional public advisers with lived experience in relevant areas. For this study, public advisers were involved in prioritising, designing the research and checking the analysis, in addition to being participants in the research.

Table 1: Characteristics of participants

**Age**	***N***	**Employment status**	***N***	**Gender**	***N***
30-39	*1*	Self employed	*2*	Female	*10*
40-49	*2*	Retired	*4*	Male	*3*
50-59	*5*	Not employed - carer	*2*	Not stated	*2*
60-69	*2*	Not employed - other	*5*		
70	*3*	Not stated	*2*		
Not stated	*2*				
**Ethnicity**		**Disability**	***N***		
White – British	*9*	Yes	*5*		
Black African and White	*1*	No	*6*		
Pakistani	*3*	Prefer not to say	*2*		
Not stated	*2*	Not stated	*2*		

Procedure

A link to the diary template (created on Qualtrics online survey software) was emailed to the participants (hereafter referred to as diarists) each Monday for 8 weeks from 20th April 2020. A Microsoft Word version was also available. The diary format used a combination of closed questions which enabled diarists to rate their general and emotional health and quality of life each week and free text for them to record, as they chose, their daily activities and general reflections about their current situation. Diarists were not routinely asked specific questions about the pandemic: the exception was in week 7 when they were asked to reflect on major change in lockdown rules.

Each diarist was paired with a researcher (EH, KK, FW, PW) for an induction session and the weekly calls. The conversation during the telephone or video calls, which lasted approximately 30 minutes, was guided by a proforma which included prompts about general wellbeing and any standout events during the week. Calls were not recorded but notes taken and these were included in the analysis. At the end of the diary period, diarists took part in a focus group which explored their experience of the diary method and participating in the study. To enable diarists to speak openly, one focus group (95 minutes) was facilitated by researchers not involved in the weekly calls (VH, GS). There were 11 participants.

Analysis

The data from 115 diary-weeks, fieldnotes from 114 calls and the focus group transcript were analysed using NVIVO-12 to organise the data. (EH, VH). An inductive thematic analysis was used to organise the whole dataset according to patterns identified by the coders (FW, EH) and to draw out similarities and differences across participants' experiences[16]. One of

For peer review only

For peer review only - http://bmjopen.bmj.com/site/about/guidelines.xhtml

the prominent themes was the diarists' observations of and role in community responses to the pandemic. To explore this aspect of the data in a more structured and focused way we conducted a deductive analysis[17] applying Chaskin's framework[18] (FW) and narrative techniques to the analysis of data coded to all relevant themes in our dataset (namely community support and responses; social networks; and volunteering). To establish trustworthiness of the findings[19], the research team met to discuss the analysis and diarists participated in a workshop to discuss preliminary findings[20,21]. Quotations used here to illustrate findings are referenced using the diarist's unique ID (1-16) and the diary week (1-8). Where data comes from weekly calls, this is stated (e.g. *Diarist5-Week1-call*).

RESULTS

The analysis of the experiences and observations of the diarists on the theme of community responses are described below as they relate to the dimensions of the Chaskin framework (Figure 1). The framework itself has been reorganised to lead with the 'conditioning influences' as the timing and purpose of the study meant that the lockdown context was the critical circumstance.

For peer review only

Figure 1. Chaskin framework dimensions applied to Diary Project findings

For peer review only

For peer review only - http://bmjopen.bmj.com/site/about/guidelines.xhtml

Conditioning influences: the lockdown context
Conditioning influences (dimension 1) are the *"mediating circumstances that may facilitate or inhibit community capacity and efforts to build it"* (18 p299). Although the Diary Study participants lived in different neighbourhoods, there were common factors that encouraged and inhibited the development of community capacity during the first lockdown.

Residential stability and activity close to home

Diarists described the way that the lockdown resulted in the adoption new routines closer to home. Under national rules people were permitted to exercise daily, and this was seen as an opportunity for social connectivity, particularly for those who lived alone. Several diarists said there were more people walking locally, resulting in additional contacts and casual conversations. As one diarist wrote, 'w*hen I take my daily exercise, people are being more friendly and actually saying hello and even stopping to have a bit of chat*' (Diarist10-week1). During a catch-up call, diarists said they were *'uplifted'* by such greetings and people acknowledging one another. For some, religious occasions which could not be celebrated in the usual way also provided an opportunity for neighbours to connect: one diarist described that during the festival of Shavuot, a neighbour celebrated by sharing food with others on the street (Diarist2-week6) and similar activity was reported during Ramadan.

There was, however, less of a sense of community for some because of where they lived. Two diarists who were also shielding described their neighbourhood as consisting of larger houses with older neighbours who rarely saw each other and another lived in a rural location, out of sight of other houses.

Personal circumstances
Changes in routine, the furlough scheme and the lack of alternative activities created human capacity and facilitated local community engagement, illustrated by one diarist who said that the suspension of their previous volunteering roles had *'led them to find other things to do to support people'* (Diarist1-Week1-call).

Some diarists, however, were not able to undertake volunteering activities that were previously '*a big part'* (Diarist6-Week5-call) of their lives and described the subsequent sense of loss. This was particularly the case for those who were shielding. The change from their normal activities was especially difficult as one diarist wrote:

> *"Inclusion is the biggest value - we are a couple with careers based on helping people be at their best. But now can't be part of this... The sense of being part of a community and not being able to contribute when you would normally be in that role - it is frustrating - feel a bit jealous not being part of the community coming together"* (Diarist4-Week1).

At the end of the study, they were still feeling restricted and a sense of isolation:

> *"I love getting 'stuck into' work and doing things - nearly always for others. I am finding it exceptionally hard not to be able to do this being shielded. I had to call on a local volunteer to get my prescriptions from the chemist and whilst it was wonderful to receive the help it also made me more frustrated that I wasn't the one doing this!"* (Diarist4-Week8).

For peer review only - http://bmjopen.bmj.com/site/about/guidelines.xhtml

Community capacity: underlying characteristics

Chaskin describes elements underlying the development of community capacity (dimension 2), including a sense of community, committed individuals, the ability to solve problems and access to resources.

Opportunities to connect and observe

The Diary Study findings suggest that during the early lockdown there was, in general, a growing sense of connectedness. This was often a physical occurrence, facilitated by socially distanced contact outdoors, but also happening online and via telephone calls. One diarist, for example, reflected on the response when a funeral precession passed along their street saying '*it brought everyone out to pay respects as it passed. It's unusual for the place to be so busy during the day and it showed the sense of community spirit that has emerged'*: they felt that this indicated *'how much community spirit there was in the locality which people hadn't realised'* (Diarist1-week1-call).

Several diarists wrote that more frequent conversations between neighbours had resulted in a greater level of understanding about the needs of people at risk in their communities. One diarist, for example, said that when they were delivering food parcels '*… many residents are now asking for other help/support/advice, TV licenses, Utility bill payments etc'* (Diarist11-Week2) and another described how a WhatsApp group had '*been a way to identify/support people perceived to be at risk on an informal basis, such as a group member with mental health'* (Diarist1-Week1-call). One diarist described how their father's neighbours had responded during the lockdown:

> *"My dad has always had good neighbours but now they have become fantastic...* [they] *have joined together to make sure all the old and vulnerable people are supported both emotional and practically"* (Diarist9-week4).

For several diarists, however, the perceptions of a mutuality of circumstances faded as the lockdown progressed. Towards the end of the study, some expressed frustration at the response of neighbours when restrictions were being lifted, which arguably worked against a sense of community at this point.

Individuals committed to problem solving

The majority of the diarists were regularly involved in some sort of community activity with a small number engaged almost daily in community responses. During the early weeks of the lockdown, organisations often suspended support services and many people, particularly those who were shielding, were isolated and struggled to get obtain essentials such as food shopping. It was clear that despite any personal difficulties' diarists were contending with, many were still thinking about maintaining contact and providing practical help to others in their community. In several cases, they supported people they had met through previous volunteer roles: one '*buddied up'* for walks in the park and another took things to someone whilst they were in hospital and checked their house.

Several diarists described their continued involvement with third sector organisations that they volunteered with and the sense of purpose these activities provided. Many activities had ceased because face-to-face contact was not an option but during the period covered by the diary project, some were re-configured and moved online. When the pilot call for a support

For peer review only - http://bmjopen.bmj.com/site/about/guidelines.xhtml

group went well, one diarist described it as *'great news!'* When they also received an email asking if they were interested in helping with a new telephone support line they *'signed up straight away and awaited my first client for a phone call on Thursday'* (Diarist1-Week3).

Access to means and resources

The availability of resources such as telephones and the internet facilitated the development of community capacity. Despite physical restrictions for some, diarists described telephone calls with isolated friends, family and neighbours. Another common theme was the way that diarists adapted, using forms of communication that were new to them, such as Zoom, Microsoft Teams and What's App to maintain existing connections and engage in new activities such as religious services, fundraising, individual and street level support, and volunteering activities.

Diarists also described the additional resources that were made available to support community initiatives including donations from individuals, businesses, and social enterprises and in one case, local authority funds. One diarist involved in a community response, for example, described donations of food from local restaurants, meals being prepared in a school kitchen and resources being gathered and packaged in space made available by a community hub (Diarist11-Week1). Others referred to restaurants and cafés delivering to vulnerable people (Diarist10-Week1) and community hampers for a local nursing home (Diarist14-Week1).

Actors, activities, and strategies: delivering the community response

Strategies are the means through which capacity is built or engaged (dimension 3). Chaskin suggests these often include a combination of community organising, organisational and leadership development or through fostering collective relationships amongst organisations. Strategies may operate through informal social processes, organised community processes or formal targeted efforts and, to convert these strategies into action, community capacity is engaged through agency stemming from individuals, networks, and organisations (dimension 4). The 'intent' of the community capacity building is described as the function (dimension 5).

Individual acts

Although there was a degree of strategic development, the most frequently reported approaches to community capacity building operated through informal interactions with individual residents proactively applying knowledge, skills, and resources. Ten diarists described the instrumental and emotional support they provided (or observed others providing), often to those who were elderly or shielding. Throughout the eight-week study, there were frequent descriptions of *'people helping each other out'* (Diarist1-Week3-call). Activities or functions included practical tasks (e.g. shopping, fetching prescriptions and delivery medicines) and providing support through socially distanced walks and telephone calls.

Informal networks

Community capacity was also developed through networks of relationships with neighbourhood groups and organisations acting together. Several diarists described their membership of social media groups which provided a forum for requests and offers of help

For peer review only - http://bmjopen.bmj.com/site/about/guidelines.xhtml

from one resident to another. One diarist described how they identified a need for this and set up a street-level WhatsApp group, saying they were *'enjoying being able to support my street through social media. There is always something to offer from shopping to advice'* (Diarist 14-Week1). Another diarist was heavily involved in a response co-ordinated by a town council. This approach was enabled by local government funding and individuals who were already active in their community mobilised social enterprises, businesses, and other local service providers to provide essential shopping and meals to residents at risk. Their local knowledge, ability to act quickly and being known in the community were seen to be essential components underlying their rapid community response (Diarist11-Week1-call).

The contribution of organisations

At an organisational level, Chaskin suggested that the development of community capacity *"might be reflected in the ability of such organizations to carry out their functions responsively, effectively, and efficiently as part of the larger system of actors and processes to which they are connected, within and beyond the community"* (18 p.298). Positive reporting of organisational community capacity building in the diaries largely focused on organisations adapting support service to be delivered online and there was enthusiasm when, a few weeks after the commencement of lockdown, diarists were able to engage in volunteering roles. This included one diarist who became very actively involved in an initiative to support people with mental health problems and a befriending project. There was also sympathy expressed for the response of professionals such as general practitioners and the clergy who were seen to be *'reaching out a much as they can but they can't fill the gaps'* (Diarist4-Week8).

But during the initial stages of lockdown, several diarists expressed the view that the needs of vulnerable residents were most frequently being met by individuals or community networks. Diarists expressed concern that organisations needed to find alternative ways to provide for service users reliant on them for social contact and support (e.g. Diarist1-Week2-call) and several suggested that organisations had often been slow to respond to the needs of the community or provide guidance to inform community-based action. Frustration was expressed about what was seen as the lack of effective response from *'well paid people'*: one diarist described this as *'a disconnect between 'grassroots' organisations and individuals doing stuff, and… councils and others talking about the doing'* (Diarist11-Week1-call) and another wrote about a lack of co-ordination when they did react:

> *"…there are numerous voluntary organisations that do not like working with each other, they seem to have lost focus as to why they exist. They all share one thing in common, they have definitely forgotten that they exist to serve and support the community and if an individual organisation can't provide that support, then signpost them to one which can"* (Diarist10-Week2).

For peer review only - http://bmjopen.bmj.com/site/about/guidelines.xhtml

It was also suggested that in some cases, organisations were unable to utilise potential community action because of pre-existing organisational arrangements, thus inhibiting the potential impact of more informal groups. One diarist reported that statutory funding was not available to community groups who were ready to respond to local need because they were not formally constituted. This was at a time when some organisations which could have been part of the community network had furloughed staff, so restricting the availability of people in key roles.

Outcomes of the increased community capacity

The outcome of the community activity (dimension 6) as identified and described by the diarists included an increase in community capacity (dimension 2) as well as outcomes relating to the tasks undertaken.

Maintaining wellbeing

Maintaining the wellbeing of residents at risk was highlighted as a motivation for the community responses. This support was clearly valued as one diarist wrote:

> *"People are mentioning that they will really miss us tomorrow!!! It's a very humbling feeling, knowing that a small meal, a chat and a smile can mean so much to someone…"* (Diarist11-Week3)

Several diarists also described the importance to their personal wellbeing of being engaged in community or volunteering work at such a challenging time. They described these activities as *'giving a sense of purpose'* (Diary3 wk2) and enhancing feeling of self-worth through being *'useful' 'of value'* and *'productive'* (Diarist11-Week1). Another diarist described the mutual benefit of being involved in a new support initiative:

> *"Had my first Check in and chat call today, went brilliantly on the phone for an hour and I think we both got something out of the experience. Look forward to my next chat next week"* (Diarist1-Week3).

But this activity also had personal physical and mental consequences. One diarist was heavily involved in the mobilisation of community network which collected, processed, and delivered food to vulnerable residents described how demanding it could be:

For peer review only - http://bmjopen.bmj.com/site/about/guidelines.xhtml

> "*Over at* [locality2] *primary school to collect pasta/soups to package and distribute later today. 11.00am, 120 food/sandwiches packages ready to load onto van, the list is growing, some residents ask to be removed as they have adequate support networks, but for every 1 we take off, 4 more ask for support. Finished delivering food around 5.45pm, really tired*" (Diarist11-Week2).

In addition to being active and engaged in proving support to others, diarists were likewise experiencing the pandemic and were dealing with the psychological impact of this. This diarist also noted the additional strain associated with being out in the community and having contact with vulnerable people:

> *"Just been told that today, will be our final day of delivering support as part of the* [organisation] *COVID-19 community response. Such a mixture of feelings. Relieved, and happy that I (along with 10 of my friends and colleagues) have so far survived contracting the virus, despite placing ourselves (and our loved ones) in danger. The stress of being in close proximity with vulnerable residents, many of them elderly, with multiple and complex health conditions has been enormous at times"* (Diary11-Week7).

New connections and information sharing

The overall perception from the diarists was that the increased contact between residents had resulted in the development of new networks and channels of communication. In turn this had led to a greater awareness of the needs of individuals within the community, information sharing and pathways for individuals and businesses to '*help out more'*. Diarists also described, through their own volunteering experiences, the new initiatives and ways of working developed by the organisations they were connected with to meet the support needs of their service users.

Longevity of the community capacity

The nature of the Diary Study, in gathering the perceptions of 15 individuals during the early weeks of the lockdown, meant it was not possible to comment on the extent to which the community capacity built or other outcomes were sustained beyond the diary period. Even during the period covered by the Diary Study, activities were changing and in one instance, a community response that had regularly delivered shopping and meals to more than 100 people was being '*wound down'*. In part this was an apparent reaction to fears that they were *'creating dependency'* but also with the understanding that furloughed staff were returning to work and there was some return to normality.

For peer review only - http://bmjopen.bmj.com/site/about/guidelines.xhtml

But several diarists, as illustrated below, did express their hope that the greater sense of community and capacity built would be sustained:

> *"Making sure we don't lose the positives There have been so many positive things that have come from the Covid situation. So much connectivity between people, huge numbers offering help, old friendships renewed, new friends being made, lots of creative solutions being identified to new and difficult problems. I really hope a lot of these really beneficial things can be captured and encouraged to still remain in place when life gets a bit more back to normal"* (Diarist4-Week8).

There was a hope that this enthusiasm could be captured but clearly also some doubt – with reference to the community connectedness they had observed, this diarist said '*What a shame we can't bottle it!'* (Diary4-Week3).

CONCLUSIONS

The research reported here aimed to explore community responses to the COVID-19 pandemic. Whilst the data from this Diary Study focused on emerging community responses rather than planned initiatives, Chaskin's exploration of community capacity proved to be a useful framework to understand the observations and experiences of the diarists during the early weeks of the first UK COVID-19 lockdown. Though actions were largely reactive, the findings did indicate that capacity building took place: participants described a sense of community, and a desire to be actively engaged; the generation of ideas and solutions to local issues as they were identified and harnessing of resources were apparent in data from diary entries and weekly calls.

Using Chaskin's framework also highlights the importance of conditioning influences and in the predicament created by the COVID-19 lockdown, the accounts of the diarists elucidating the critical role of context in the development of community capacity. Inhibiting influences include the lockdown rules, restrictions on people who were shielding and the absence of existing services and other sources of support. The diarists described how community capacity building was supported by people's physical proximity to home, the use of information technology, greater availability, and by goodwill which frequently appeared to engender a feeling of being 'in it together'.

The picture of the community response presented by the diaries contains insights into different population characteristics that could inform the recovery. It supports research which suggests that residents must be engaged if more systemic recovery support is to be valued e.g.(22) and reveals valuable information from members of the community about the knowledge and processes that resulted in the provision of essential support whilst there were gaps in service delivery. As the National Institute for Health and Care Excellence has indicated, the recovery period provides an opportunity for local government and health and

For peer review only - http://bmjopen.bmj.com/site/about/guidelines.xhtml

care services to build on the strengths of communities to ensure initiatives are 'relevant and meaningful'(23).

The main functions of the community response described by the diarists was to provide essential resources to residents at risk, and to a lesser extent, social support. The sharing of intelligence was also required to enable this to happen, a finding in line with other work which described the *'creative solutions'* developed by residents who understand their community(24) Whilst diarists wrote about the communication of lockdown rules and government health messages, there was no evidence that this formed any part of their activities, contrasting with the type of activities described in previous epidemics(7). This finding supports the UK Government's research that found informal volunteers were most likely to be employed maintaining social contact with less mobile neighbours and carrying out practical tasks such as shopping and collecting medicine(12).

Support was provided through community organising, primarily via individual agency and local connections and, to a lesser extent, organised community processes. This resonates with other research findings(5) where the main actors in community pandemic responses were community leaders, community and faith-based organisations, community networks, committees, and individuals. Some diarists referred to targeted efforts of larger organisations but in general the activities described were not part of a strategic approach, rather they were informal and evolving in response to the initial crisis and rapidly changing circumstances. The prominence of these individual and self-organised street-level activities appeared to be effective for those residents who were identified: practical support was provided at a time when formal support services were not available or not able to cope. Further supporting the findings of Gilmore et al.(5) there was no discussion in the diaries about community involvement in wider strategic decision-making.

The greater community capacity generated during the time covered by the Diary Study appears to have been created through a wider awareness of need within the community and the development of channels to share this information and respond to need (e.g. through social media groups). Other outcomes include access to new sources of support such as co-walking and befriending which enabled the wellbeing of residents at risk to be maintained. It is also important to acknowledge the personal toll for those involved in a community response: volunteers were similarly experiencing the lockdown with its associated strain on themselves, their families, and acquaintances in addition to experiencing anxieties about their contact with infected and clinically vulnerable people. This raises questions about appropriate support and the resources required to sustain successful elements of community-based responses during the COVID-19 recovery and beyond.

In contrast with other community action frameworks, the concepts of empowerment and control do not feature prominently in Chaskin's dimensions of community capacity building. Similarly, the processes and outcomes described by the diarists did not include references to feelings of empowerment and control. One apparent reason for this may be that the unique context generated by the COVID-19 lockdown meant that the singular concern of many individuals, networks and organisations was to meet the immediate needs of residents most at risk. Consequently, in this context, Chaskin's framework was useful to describe this *'inward gaze'*(13) with its focus on, for example, community capacities, and neighbourhood conditions. But moving forward, and beyond the COVID-19 recovery, it is argued that power and control need to be integral elements in the consideration of community capacity,

For peer review only - http://bmjopen.bmj.com/site/about/guidelines.xhtml

requiring an *'outward gaze'* on the political and social change required to enable structural gaps and inequalities to be addressed.

The risk of community capacity building being considered out with empowerment and control is a particular concern at a time when there are reductions in state spending and, as Popay et al.(13) argue, pressure to focus on *'equipping communities to use their 'assets' to manage 'shocks' like COVID-19—to adapt to, rather than transform, existing inequalities'* (p.8). With reduced public expenditure, process of 'citizen-shift' has also been described whereby interventions are increasingly individualised and service providers are enabled to create upstream energy from grassroots level to mobilise against inequalities(25). This is an important area for future research and could inform strategies to "build back better".

Some potential limitations of the study should be noted. Firstly, the small participant sample (n=15), consisted of an older age group and their roles as Public Advisers means that findings should be considered in the context of this study. Though they were from a range of backgrounds and with differing personal circumstances, they were all previously connected with volunteering and community networks in various ways. Second, although the findings of this study suggest that community capacity building took place, the short-term nature of the study means it is not possible to assess the extent to which it was sustained or whether there was a lasting impact. Finally, the diary entries and calls also do not provide a complete picture of activity with local communities, rather they are the observations and experiences these individuals chose to report: the method adopted meant the agenda was set by diarists and researchers did not ask diarists about any possible 'gaps' in their accounts.

There have been few qualitative diary studies completed during crisis situations [5,10,11,12] and the findings of this research suggest that the combination of written diaries and weekly calls was a useful way to gather the experiences and observations of participants during the COVID-19 lockdown. The study also contributes to building wider knowledge around community responses in the COVID-19 pandemic.

The findings illuminate the community responses experienced and observed at a time of considerable uncertainty and threats to wellbeing resulting from the COVID-19 pandemic. Chaskin's community capacity building framework focused analytical attention on the conditional influences and underlying characteristics of community capacity in addition to the strategies, actors, intent, and outcomes. This demonstrated the complexity of the situation and the central importance of the specific context and of individual agency: residents were engaged (sometimes intensively) in developing links, solutions, working with what they had and, in some cases, with organisations that were in a position to respond. This confirms the likelihood that many communities could be left behind if there were not equipped individuals and/or community networks able fill the gaps in the organisational response, at least in the short-term. This could lead to increased health inequalities.

A number of directions for future research have emerged from the study. These include insights from residents who began their volunteering journey during the pandemic and looking at the impact of the community response on the wellbeing of those involved as well as those being supported. Investigating whether individuals and community networks involved in the immediate response were mobilised in the recovery post pandemic would be valuable: embedding an *'outward gaze'* into future research could also ascertain the extent to which community empowerment and involvement in wider decision-making results in strategic dimension to community capacity building post COVID-19. (5) Identifying

For peer review only - http://bmjopen.bmj.com/site/about/guidelines.xhtml

commonalities or differences in experiences across communities could also provide valuable information about inhibiting influences for 'communities left behind. In addition, this could also help us to explore relationships and networks that have been the most powerful in responding to local needs.

FUNDING

This research was funded by the National Institute for Health Research (NIHR) Applied Research Collaboration North West Coast (ARC-NWC). The views expressed are those of the author(s) and not necessarily those of the NHS, the NIHR or the Department of Health and Social Care.

ETHICS APPROVAL

Ethics approval was granted by Lancaster University Faculty of Health and Medicine's Ethics Committee in April 2020 (FHMREC19076).

CONFLICT OF INTERESTS

The author(s) declared no potential conflicts of interest with respect to the research, authorship and/or publication of this article.

AUTHOR CONTRIBUTION

Fiona Ward designed the study, developed the protocol, co-ordinated the data collection, analysed the data, wrote the manuscript; Emma Halliday designed the study, collected and analysed the data, reviewed the manuscript. Vivien Holt collected the data and reviewed the manuscript; Koser Khan collected the data and reviewed the manuscript; Gill Sadler collected the data; Paula Wheeler collected the data and reviewed the manuscript; Joanna Goldthorpe wrote the manuscript and provided methodological and analytical support.

The authors wish to thank all ARC-NWC Public Advisers who participated in the study and to Alan Price and Neil Joseph who reviewed the paper. Thanks also to Olga Angulo-Judez, Lancaster University, who provided technical support and training for diarists in the use of online methods.
This paper is dedicated to the memory of Gill Sadler who sadly passed away in January 2021.

DATA AVIAILABILITY STATEMENT

The data underlying this article cannot be shared publicly due to the need to protect the privacy and confidentiality of individuals that participated in the study. The data will be shared on reasonable request to the corresponding author.

REFERENCES

1. Sobande F. 'We're all in this together': Commodified notions of connection, care and community in brand responses to COVID-19. European Journal of Cultural Studies. 2020;23(6):1033-7.
2. Bambra C, Riordan R, Ford J, Matthews F. The COVID-19 pandemic and health inequalities. J Epidemiol Community Health. 2020;74(11):964-8.
3. Mishra V, Seyedzenouzi G, Almohtadi A, Chowdhury T, Khashkhusha A, Axiaq A, et al. Health inequalities during COVID-19 and their effects on morbidity and mortality. Journal of healthcare leadership. 2021;13:19.

For peer review only - http://bmjopen.bmj.com/site/about/guidelines.xhtml

4. Marmot M, Allen J, Goldblatt P, Herd E, Morrison J. Build back fairer: the COVID-19 Marmot review. The pandemic, socioeconomic and health inequalities in England. 2020.
5. Gilmore B, Ndejjo R, Tchetchia A, De Claro V, Mago E, Lopes C, et al. Community engagement for COVID-19 prevention and control: a rapid evidence synthesis. BMJ global health. 2020;5(10):e003188.
6. Hocevar KP, Metzger, M., & Flanagin, A. J. Source Credibility, Expertise, and Trust in Health and Risk Messaging. Oxford Research Encyclopedia of Communication: Oxford University Press; 2017.
7. Ghio D, Lawes-Wickwar S, Tang MY, Epton T, Howlett N, Jenkinson E, et al. What influences people's responses to public health messages for managing risks and preventing infectious diseases? A rapid systematic review of the evidence and recommendations. 2020.
8. Haines A, de Barros EF, Berlin A, Heymann DL, Harris MJ. National UK programme of community health workers for COVID-19 response. The Lancet. 2020;395(10231):1173-5.
9. South J, Stansfield J, Amlôt R, Weston D. Sustaining and strengthening community resilience throughout the COVID-19 pandemic and beyond. Perspectives in Public Health. 2020;140(6):305-8.
10. Mak HW, Fancourt D. Predictors of engaging in voluntary work during the Covid-19 pandemic: analyses of data from 31,890 adults in the UK. Perspectives in Public Health. 2020:1757913921994146.
11. Carlsen HB, Toubøl J, Brincker B. On solidarity and volunteering during the COVID-19 crisis in Denmark: the impact of social networks and social media groups on the distribution of support. European Societies. 2021;23(sup1):S122-S40.
12. Department of Digital C, Media & Sport & Office for Civil Society. A Look at Volunteering during the Response to COVID-19. 2021. A look at volunteering during the response to COVID-19 2021.
13. Popay J, Whitehead M, Ponsford R, Egan M, Mead R. Power, control, communities and health inequalities I: theories, concepts and analytical frameworks. Health promotion international. 2020.
14. Chaskin RJ. Building community capacity: A definitional framework and case studies from a comprehensive community initiative. Urban affairs review. 2001;36(3):291-323.
15. Medd W, Deeming H, Walker G, Whittle R, Mort M, Twigger-Ross C, et al. The flood recovery gap: a real-time study of local recovery following the floods of J une 2007 in H ull, N orth E ast E ngland. Journal of Flood Risk Management. 2015;8(4):315-28.
16. Mort M, Convery I, Baxter J, Bailey C. Psychosocial effects of the 2001 UK foot and mouth disease epidemic in a rural population: qualitative diary based study. Bmj. 2005;331(7527):1234.
17. Herron R, Dansereau L, Wrathall M, Funk L, Spencer D. Using a flexible diary method rigorously and sensitively with family carers. Qualitative health research. 2019;29(7):1004-15.
18. Braun V, Clarke V. Using thematic analysis in psychology. Qualitative research in psychology. 2006;3(2):77-101.
19. Srivastava A, Thomson SB. Framework analysis: a qualitative methodology for applied policy research. 2009.
20. Peters S. Qualitative research methods in mental health. Royal College of Psychiatrists; 2010.
21. Birt L, Scott S, Cavers D, Campbell C, Walter F. Member checking: a tool to enhance trustworthiness or merely a nod to validation? Qualitative health research. 2016;26(13):1802-11.
22. Harkins C. Supporting community recovery and resilience in response to the COVID-19 pandemic–a rapid review of evidence. Glasgow; 2020.
23. Maiden H, Jagroo J, Shearn P, Wasielewska A, Nolan K, Baillie N. Spotlight on community engagement: NICE resources in the context of COVID-19—NICE public health guidance update. Journal of Public Health (Oxford, England). 2021.
24. McMillan R. Learning from nine months of crisis response: Local Trust; 2020 [Available from: https://localtrust.org.uk/news-and-stories/blog/learning-from-nine-months-of-crisis-response/

For peer review only

For peer review only - http://bmjopen.bmj.com/site/about/guidelines.xhtml

426 25. Williams O, Fullagar S. Lifestyle drift and the phenomenon of 'citizen shift'in contemporary UK health policy. Sociology of health & illness. 2019;41(1):20-35.

428
429
430
431

For peer review only

For peer review only - http://bmjopen.bmj.com/site/about/guidelines.xhtml

Figure 1. Chaskin framework dimensions applied to Diary Project findings

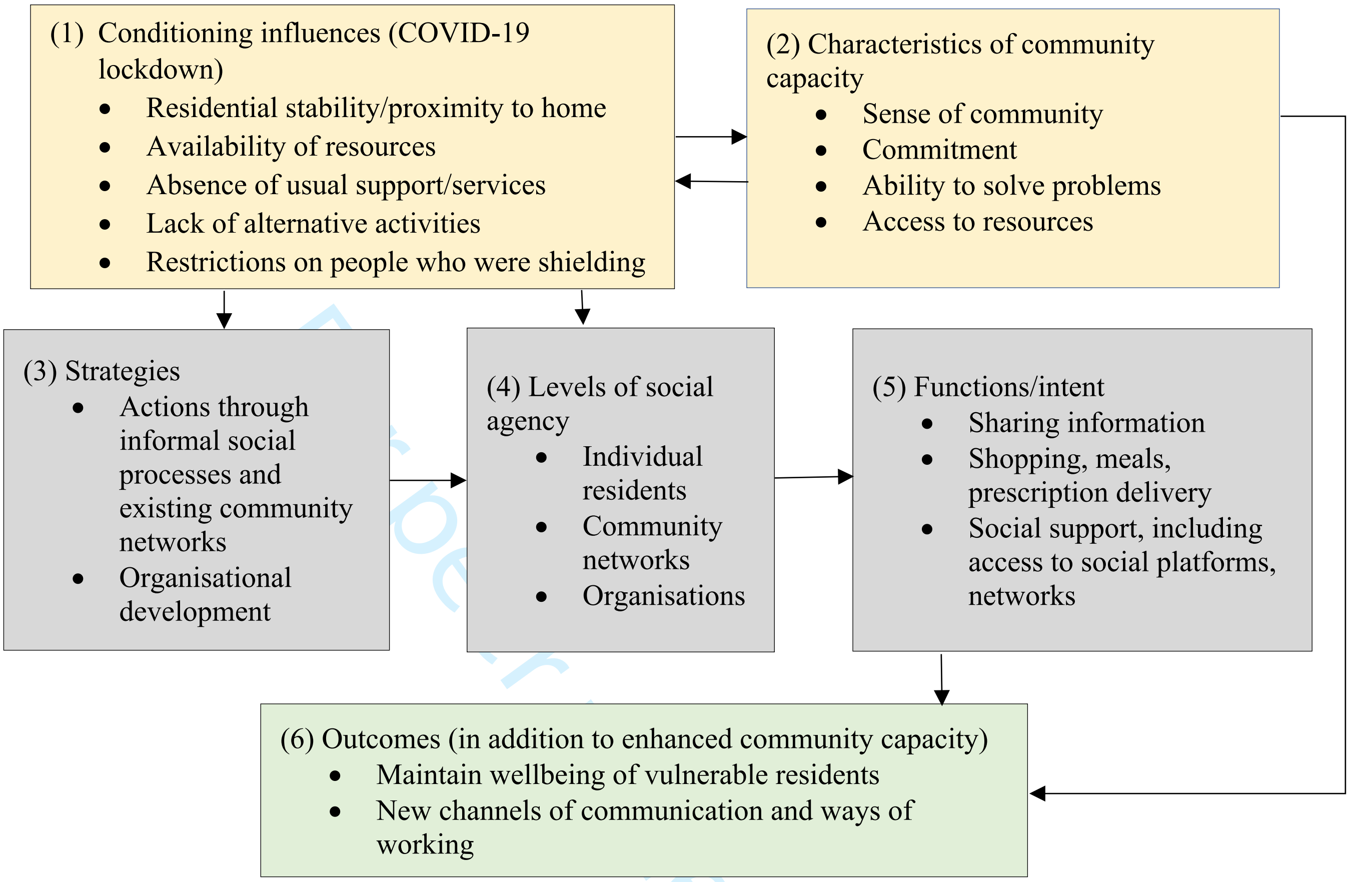

For peer review only

For peer review only - http://bmjopen.bmj.com/site/about/guidelines.xhtml

COREQ (COnsolidated criteria for REporting Qualitative research) Checklist

A checklist of items that should be included in reports of qualitative research. You must report the page number in your manuscript where you consider each of the items listed in this checklist. If you have not included this information, either revise your manuscript accordingly before submitting or note N/A.

Topic	Item No.	Guide Questions/Description	Reported on Page No.
**Domain 1: Research team and reflexivity**			
Personal characteristics			
Interviewer/facilitator	1	Which author/s conducted the interview or focus group?	
Credentials	2	What were the researcher's credentials? E.g. PhD, MD	
Occupation	3	What was their occupation at the time of the study?	
Gender	4	Was the researcher male or female?	
Experience and training	5	What experience or training did the researcher have?	
Relationship with participants			
Relationship established	6	Was a relationship established prior to study commencement?	
Participant knowledge of the interviewer	7	What did the participants know about the researcher? e.g. personal goals, reasons for doing the research	
Interviewer characteristics	8	What characteristics were reported about the inter viewer/facilitator? e.g. Bias, assumptions, reasons and interests in the research topic	
**Domain 2: Study design**			
Theoretical framework			
Methodological orientation and Theory	9	What methodological orientation was stated to underpin the study? e.g. grounded theory, discourse analysis, ethnography, phenomenology, content analysis	
Participant selection			
Sampling	10	How were participants selected? e.g. purposive, convenience, consecutive, snowball	
Method of approach	11	How were participants approached? e.g. face-to-face, telephone, mail, email	
Sample size	12	How many participants were in the study?	
Non-participation	13	How many people refused to participate or dropped out? Reasons?	
Setting			
Setting of data collection	14	Where was the data collected? e.g. home, clinic, workplace	
Presence of non-participants	15	Was anyone else present besides the participants and researchers?	
Description of sample	16	What are the important characteristics of the sample? e.g. demographic data, date	
Data collection			
Interview guide	17	Were questions, prompts, guides provided by the authors? Was it pilot tested?	
Repeat interviews	18	Were repeat inter views carried out? If yes, how many?	
Audio/visual recording	19	Did the research use audio or visual recording to collect the data?	
Field notes	20	Were field notes made during and/or after the inter view or focus group?	
Duration	21	What was the duration of the inter views or focus group?	
Data saturation	22	Was data saturation discussed?	
Transcripts returned	23	Were transcripts returned to participants for comment and/or	

For peer review only

Topic	Item No.	Guide Questions/Description	Reported on Page No.
		correction?	
**Domain 3: analysis and findings**			
Data analysis			
Number of data coders	24	How many data coders coded the data?	
Description of the coding tree	25	Did authors provide a description of the coding tree?	
Derivation of themes	26	Were themes identified in advance or derived from the data?	
Software	27	What software, if applicable, was used to manage the data?	
Participant checking	28	Did participants provide feedback on the findings?	
Reporting			
Quotations presented	29	Were participant quotations presented to illustrate the themes/findings? Was each quotation identified? e.g. participant number	
Data and findings consistent	30	Was there consistency between the data presented and the findings?	
Clarity of major themes	31	Were major themes clearly presented in the findings?	
Clarity of minor themes	32	Is there a description of diverse cases or discussion of minor themes?	

Developed from: Tong A, Sainsbury P, Craig J. Consolidated criteria for reporting qualitative research (COREQ): a 32-item checklist for interviews and focus groups. *International Journal for Quality in Health Care*. 2007. Volume 19, Number 6: pp. 349 – 357

Once you have completed this checklist, please save a copy and upload it as part of your submission. DO NOT include this checklist as part of the main manuscript document. It must be uploaded as a separate file.

For peer review only

BMJ Open

How did communities in North West England respond to the COVID-19 lockdown? Findings from a diary study

Journal:	*BMJ Open*
Manuscript ID	bmjopen-2021-057774.R2
Article Type:	Original research
Date Submitted by the Author:	05-Sep-2022
Complete List of Authors:	Ward, Fiona; Lancaster University, Applied Research Collaboration North West Coast<br>Halliday, Emma; Lancaster University, Applied Research Collaboration North West Coast<br>Holt, Vivien; Lancaster University, Applied Research Collaboration North West Coast<br>Khan, Koser; Lancaster University, Applied Research Collaboration North West Coast<br>Sadler, Gill; Lancaster University, Applied Research Collaboration North West Coast<br>Wheeler, Paula ; Lancaster University, Applied Research Collaboration North West Coast<br>Goldthorpe, Joanna; Lancaster University, Applied Research Collaboration North West Coast
**Primary Subject Heading**:	Public health
Secondary Subject Heading:	Qualitative research, Health policy, Research methods, Mental health
Keywords:	COVID-19, PUBLIC HEALTH, QUALITATIVE RESEARCH

SCHOLARONE™
Manuscripts

BMJ

I, the Submitting Author has the right to grant and does grant on behalf of all authors of the Work (as defined in the below author licence), an exclusive licence and/or a non-exclusive licence for contributions from authors who are: i) UK Crown employees; ii) where BMJ has agreed a CC-BY licence shall apply, and/or iii) in accordance with the terms applicable for US Federal Government officers or employees acting as part of their official duties; on a worldwide, perpetual, irrevocable, royalty-free basis to BMJ Publishing Group Ltd ("BMJ") its licensees and where the relevant Journal is co-owned by BMJ to the co-owners of the Journal, to publish the Work in this journal and any other BMJ products and to exploit all rights, as set out in our licence.

The Submitting Author accepts and understands that any supply made under these terms is made by BMJ to the Submitting Author unless you are acting as an employee on behalf of your employer or a postgraduate student of an affiliated institution which is paying any applicable article publishing charge ("APC") for Open Access articles. Where the Submitting Author wishes to make the Work available on an Open Access basis (and intends to pay the relevant APC), the terms of reuse of such Open Access shall be governed by a Creative Commons licence – details of these licences and which Creative Commons licence will apply to this Work are set out in our licence referred to above.

Other than as permitted in any relevant BMJ Author's Self Archiving Policies, I confirm this Work has not been accepted for publication elsewhere, is not being considered for publication elsewhere and does not duplicate material already published. I confirm all authors consent to publication of this Work and authorise the granting of this licence.

For peer review only

Title: How did communities in North West England respond to the COVID-19 lockdown? Findings from a diary study

Fiona Ward[1], Emma Halliday[1], Vivien Holt[1], Koser Khan[1], Gill Sadler[1], Paula Wheeler[1] Joanna Goldthorpe[1, 2]

[1]Division of Health Research, Faculty of Health & Medicine, Lancaster University, Lancaster, LA1 4YG.

[2] Corresponding author

Address for corresponding author

Division of Health Research, Faculty of Health & Medicine, Lancaster University, Lancaster, UK, LA1 4YG.

Email: j.goldthorpe@lancaster.ac.uk

For peer review only

ABSTRACT

OBJECTIVES: During the COVID-19 pandemic the UK government and public health leaders advocated for community level responses to support vulnerable people. This activity could be planned and co-ordinated, however much was informal and developed organically. The effects on the individuals who were involved in providing and receiving informal support and implications for their communities has not been widely explored. The aim of this study was therefore to document and explore the nature, potential effects and longevity of community responses to the COVID-19 pandemic. PARTICIPANTS: We asked 15 individuals in North West England to keep a diary during the first UK COVID-19 lockdown. Over 8 weeks, diaries were completed and supported with weekly calls with researchers. A community capacity building framework was used to explore reported community responses to the Covid pandemic. RESULTS: Diarists described community characteristics that enabled and hindered helpful responses in the lockdown context. Diarists frequently described informal approaches with residents acting alone or with near neighbours, although there were examples of community networks and residents recommencing formal volunteering activities. Diarists reported communities providing practical help and social support to vulnerable people. Participants perceived a greater sense of community, increased contact between residents and new networks during the period covered. CONCLUSION: The diaries provided valuable insights and the framework was a useful tool to explore the COVID-19 lockdown context. The findings indicate that organic capacity building took place, primarily via individual agency, highlighting the risk of communities being 'left behind' if there were not individuals or community networks available with resources to plug gaps in organisational support. Recommendations to sustain helpful responses to the pandemic include further consideration of ongoing community mobilisation, empowerment and community control within the capacity building framework.

Strengths and limitations of this study

- The study's diary methodology allowed for data to be collected in real time during the first Covid-19 pandemic lockdown, capturing authentic, in-the-moment insight from participants.
- The findings were organised around an established framework (Chaskin's framework of community capacity building).
- The findings reflected the experiences of an older, mostly female experience with previous experience of volunteering and/ or community service.

INTRODUCTION

Reaching and supporting people at risk, particularly those living in disadvantaged communities has been vital to containing the effects of the COVID-19 pandemic and reducing associated morbidity and mortality. Despite government and commercial organisations declaring that "we are all in it together"(1), not everyone experienced the pandemic in the same way due to the effects of national and international structural and systemic inequalities. The effects of the COVID-19 pandemic are being felt particularly

For peer review only - http://bmjopen.bmj.com/site/about/guidelines.xhtml

severely by clinically vulnerable individuals and globally along racial, ethnic, and socio-economic divides(2-4). These disproportionate effects highlight and exacerbate the damaging effect of health inequalities that existed before the pandemic and influenced the publication of the Marmot review(4). This report examines the reasons behind the UK's status as having one of the highest COVID-19 mortality rates in Europe and describes how we can "build back fairer" by introducing polices that reduce health inequalities.

Research from previous infectious disease pandemics indicates that smaller scale social and community responses may be especially important in reaching vulnerable and marginalised populations. A rapid review of research into community responses to recent pandemics including Ebola, SARS, Middle East Respiratory Syndrome and H1N1(5) suggest that they may be crucial in engendering trust and goodwill in those who are suspicious of the motives of national governments and international organisations such as World Health Organisation. This trust and credibility is vital for delivering appropriate health messages(6), modelling behaviours, and establishing social norms that reduce viral transmission(7).

Public health leaders in the UK proposed and advocated for community responses to support vulnerable people during the pandemic. These included a "call to arms" for those with basic medical training to deliver frontline home support(8) and a framework for a structured 'whole systems' approaches to community-based initiatives in order to build community resilience [in addition to capacity building around virology, vaccinations and reducing viral transmission)(9). These approaches would have been unlike most identified by Gilmore et al.(5) which found community engagement in high income countries (Australia, Canada, USA) usually entailed passive involvement such as targeted consultation with minority populations and very few had an equity focus or reported informal, locally driven activity.

To date, several quantitative studies have looked at volunteering during the pandemic. They have reported on the demographics of volunteers during the early lockdown period (people who were older, non-white and not living with partner), the categories of volunteering (formal, social action and community)(10) and how they were mobilised (social networks and social media groups)(11). A survey carried out on behalf of the UK Department of Culture, Media & Sport and Office for Civil Society looked specifically at the nature of informal and formal volunteering. It found that participants volunteering informally were more likely to: keep in touch with someone who has difficulties getting out; carrying out tasks such as shopping and collecting medicine, and less likely to provide transport or escort to venues outside the home.(12) This research did not, however, explore the experiences of volunteers or others in their communities.

Gilmore et al.(5) concluded that little attention has been given to informal and unstructured responses to the COVID-19 pandemic carried out by citizens in their own localities and that future pandemic research should include a focus on documenting organic community engagement This research should additionally be of interest to readers concerned with community empowerment and place-based approaches; citizen-led community initiatives have potential to positively disrupt the power relationships inherent in top-down community development initiatives, resulting in potential benefits for public health(13). If we are to "build back better" and "fairer" it makes sense to capture, support and resource acceptable bottom-up, equitable models of community engagement that have potential to reduce health inequalities.

For peer review only - http://bmjopen.bmj.com/site/about/guidelines.xhtml

Chaskin's (2001) definition and framework for community capacity offers a useful lens through which to explore organic community responses to the recent pandemic. It provides a structure for exploration around how local resources were informally mobilised to solve neighbourhood-level problems, and the parameters of these efforts. There may be a point at which individuals and resources come together to build capacity in communities, but also a point at which this capacity is reached and more formal, planned approaches may need to be mobilised. Chaskin (2001)conceptualises community capacity as (14)
"…the interaction of human capital, organizational resources, and social capital existing within a given community that can be leveraged to solve collective problems and improve or maintain the well-being of a given community. It may operate through informal social processes and/or organized effort." (18, p.295).

The study was exploratory and sought to broadly understand public experiences of the pandemic including its effects on diarists' everyday lives. This paper uses Chaskin's Framework to present perspectives on one of the prominent themes - that of local community responses to the first COVID-19 lockdown. Our specific research question is: How did communities in North West England respond to the COVID-19 lockdown?

METHODS

Design

This study used solicited diaries, a research method employed in previous crisis situations to record personal experiences and observations(15, 16). Weekly calls complemented the written diaries as these have been found to aid diary completion and improve the quality of the information recorded(17).

Setting

The study was undertaken by researchers from the Applied Research Collaboration North West Coast (ARC-NWC), a collaboration of universities, NHS providers and commissioners, local authorities and third sector organisations which support applied research that responds to the needs of local populations and health and care systems. Public and community involvement is central to this work and the study participants were ARC-NWC public contributors or 'Public Advisers'. The participants all lived in communities based in the North Western coastal area of England.

Participants

All Public Advisers registered with ARC-NWC were invited, by email, to take part in the study. This means that the participants and researchers may have met previously. A total of 16 people expressed an interest, although one withdrew before the diaries commenced. The remaining 15 completed the 8-week study. All were aged over 18 years. Although not purposively sampled, there was diversity in the circumstances of those who took part. They included people living alone, with families, in clinical at-risk groups, shielding households and people with caring responsibilities. Characteristics of participants are shown in Table 1. Written consent was obtained from diarists, who were also reminded of their rights during each weekly call.

Patient and Public Involvement

ARC NWC has paid public advisers who work alongside researchers at each stage of the research. Each ARC NWC theme (research team) has a public adviser as a co-lead to lead on prioritisation and public involvement. Teams involved in specific research studies recruit

For peer review only - http://bmjopen.bmj.com/site/about/guidelines.xhtml

additional public advisers with lived experience in relevant areas. For this study, public advisers were involved in prioritising, designing the research and checking the analysis, in addition to being participants in the research.

Table 1: Characteristics of participants

Age	*N*	Employment status	*N*	Gender	*N*
30-39	*1*	Self employed	*2*	Female	*10*
40-49	*2*	Retired	*4*	Male	*3*
50-59	*5*	Not employed - carer	*2*	Not stated	*2*
60-69	*2*	Not employed - other	*5*		
70	*3*	Not stated	*2*		
Not stated	*2*				

Ethnicity		Disability	*N*
White – British	*9*	Yes	*5*
Black African and White	*1*	No	*6*
Pakistani	*3*	Prefer not to say	*2*
Not stated	*2*	Not stated	*2*

Procedure

A link to the diary template (created on Qualtrics online survey software) was emailed to the participants (hereafter referred to as diarists) each Monday for 8 weeks from 20th April 2020. A Microsoft Word version was also available. The diary format used a combination of closed questions which enabled diarists to rate their general and emotional health and quality of life each week and free text for them to record, as they chose, their daily activities and general reflections about their current situation. Diarists were not routinely asked specific questions about the pandemic: the exception was in week 7 when they were asked to reflect on major change in lockdown rules.

Each diarist was paired with a researcher (EH, KK, FW, PW) for an induction session and the weekly calls. The conversation during the telephone or video calls, which lasted approximately 30 minutes, was guided by a proforma which included prompts about general wellbeing and any standout events during the week. Calls were not recorded but notes taken and these were included in the analysis. At the end of the diary period, diarists took part in a focus group which explored their experience of the diary method and participating in the study. To enable diarists to speak openly, one focus group (95 minutes) was facilitated by researchers not involved in the weekly calls (VH, GS). There were 11 participants.

Analysis

The data from 115 diary-weeks, fieldnotes from 114 calls and the focus group transcript were analysed using NVIVO-12 to organise the data. (EH, VH). An inductive thematic analysis was used to organise the whole dataset according to patterns identified by the coders (FW, EH) and to draw out similarities and differences across participants' experiences[16]. One of the prominent themes was the diarists' observations of and role in community responses to

For peer review only

For peer review only - http://bmjopen.bmj.com/site/about/guidelines.xhtml

178 the pandemic. To explore this aspect of the data in a more structured and focused way we conducted a deductive analysis[17] applying Chaskin's framework[18] (FW) and narrative techniques to the analysis of data coded to all relevant themes in our dataset (namely community support and responses; social networks; and volunteering). To establish trustworthiness of the findings[19], the research team met to discuss the analysis and diarists participated in a workshop to discuss preliminary findings[20,21]. Quotations used here to illustrate findings are referenced using the diarist's unique ID (1-16) and the diary week (1-8). Where data comes from weekly calls, this is stated (e.g. *Diarist5-Week1-call*).

RESULTS

The analysis of the experiences and observations of the diarists on the theme of community responses are described below as they relate to the dimensions of the Chaskin framework (Figure 1). The framework itself has been reorganised to lead with the 'conditioning influences' as the timing and purpose of the study meant that the lockdown context was the critical circumstance.

Figure 1. Chaskin framework dimensions applied to Diary Project findings

For peer review only - http://bmjopen.bmj.com/site/about/guidelines.xhtml

Conditioning influences: the lockdown context

Conditioning influences (dimension 1) are the *"mediating circumstances that may facilitate or inhibit community capacity and efforts to build it"* (18 p299). Although the Diary Study participants lived in different neighbourhoods, there were common factors that encouraged and inhibited the development of community capacity during the first lockdown.

Residential stability and activity close to home

Diarists described the way that the lockdown resulted in the adoption new routines closer to home. Under national rules people were permitted to exercise daily, and this was seen as an opportunity for social connectivity, particularly for those who lived alone. Several diarists said there were more people walking locally, resulting in additional contacts and casual conversations. As one diarist wrote, '*when I take my daily exercise, people are being more friendly and actually saying hello and even stopping to have a bit of chat*' (Diarist10-week1). During a catch-up call, diarists said they were *'uplifted'* by such greetings and people acknowledging one another. For some, religious occasions which could not be celebrated in the usual way also provided an opportunity for neighbours to connect: one diarist described that during the festival of Shavuot, a neighbour celebrated by sharing food with others on the street (Diarist2-week6) and similar activity was reported during Ramadan.

There was, however, less of a sense of community for some because of where they lived. Two diarists who were also shielding described their neighbourhood as consisting of larger houses with older neighbours who rarely saw each other and another lived in a rural location, out of sight of other houses.

Personal circumstances

Changes in routine, the furlough scheme and the lack of alternative activities created human capacity and facilitated local community engagement, illustrated by one diarist who said that the suspension of their previous volunteering roles had *'led them to find other things to do to support people'* (Diarist1-Week1-call).

Some diarists, however, were not able to undertake volunteering activities that were previously '*a big part'* (Diarist6-Week5-call) of their lives and described the subsequent sense of loss. This was particularly the case for those who were shielding. The change from their normal activities was especially difficult as one diarist wrote:

> *"Inclusion is the biggest value - we are a couple with careers based on helping people be at their best. But now can't be part of this... The sense of being part of a community and not being able to contribute when you would normally be in that role - it is frustrating - feel a bit jealous not being part of the community coming together"* (Diarist4-Week1).

At the end of the study, they were still feeling restricted and a sense of isolation:

> *"I love getting 'stuck into' work and doing things - nearly always for others. I am finding it exceptionally hard not to be able to do this being shielded. I had to call on a local volunteer to get my prescriptions from the chemist and whilst it was wonderful to receive the help it also made me more frustrated that I wasn't the one doing this!"* (Diarist4-Week8).

Community capacity: underlying characteristics

For peer review only - http://bmjopen.bmj.com/site/about/guidelines.xhtml

Chaskin describes elements underlying the development of community capacity (dimension 2), including a sense of community, committed individuals, the ability to solve problems and access to resources.

Opportunities to connect and observe

The Diary Study findings suggest that during the early lockdown there was, in general, a growing sense of connectedness. This was often a physical occurrence, facilitated by socially distanced contact outdoors, but also happening online and via telephone calls. One diarist, for example, reflected on the response when a funeral precession passed along their street saying '*it brought everyone out to pay respects as it passed. It's unusual for the place to be so busy during the day and it showed the sense of community spirit that has emerged'*: they felt that this indicated *'how much community spirit there was in the locality which people hadn't realised'* (Diarist1-week1-call).

Several diarists wrote that more frequent conversations between neighbours had resulted in a greater level of understanding about the needs of people at risk in their communities. One diarist, for example, said that when they were delivering food parcels '*… many residents are now asking for other help/support/advice, TV licenses, Utility bill payments etc'* (Diarist11-Week2) and another described how a WhatsApp group had '*been a way to identify/support people perceived to be at risk on an informal basis, such as a group member with mental health'* (Diarist1-Week1-call). One diarist described how their father's neighbours had responded during the lockdown:

> *"My dad has always had good neighbours but now they have become fantastic...* [they] *have joined together to make sure all the old and vulnerable people are supported both emotional and practically"* (Diarist9-week4).

For several diarists, however, the perceptions of a mutuality of circumstances faded as the lockdown progressed. Towards the end of the study, some expressed frustration at the response of neighbours when restrictions were being lifted, which arguably worked against a sense of community at this point.

Individuals committed to problem solving

The majority of the diarists, both male and female, were regularly involved in some sort of community activity with a small number engaged almost daily in community responses. During the early weeks of the lockdown, organisations often suspended support services and many people, particularly those who were shielding, were isolated and struggled to get obtain essentials such as food shopping. It was clear that despite any personal difficulties' diarists were contending with, many were still thinking about maintaining contact and providing practical help to others in their community. In several cases, they supported people they had met through previous volunteer roles: one '*buddied up'* for walks in the park and another took things to someone whilst they were in hospital and checked their house.

Several diarists described their continued involvement with third sector organisations that they volunteered with and the sense of purpose these activities provided. Many activities had ceased because face-to-face contact was not an option but during the period covered by the diary project, some were re-configured and moved online. When the pilot call for a support group went well, one diarist described it as *'great news!*' When they also received an email asking if they were interested in helping with a new telephone support line they *'signed up straight away and awaited my first client for a phone call on Thursday'* (Diarist1-Week3).

For peer review only - http://bmjopen.bmj.com/site/about/guidelines.xhtml

Access to means and resources

The availability of resources such as telephones and the internet facilitated the development of community capacity. Despite physical restrictions for some, diarists described telephone calls with isolated friends, family and neighbours. Another common theme was the way that diarists adapted, using forms of communication that were new to them, such as Zoom, Microsoft Teams and What's App to maintain existing connections and engage in new activities such as religious services, fundraising, individual and street level support, and volunteering activities.

Diarists also described the additional resources that were made available to support community initiatives including donations from individuals, businesses, and social enterprises and in one case, local authority funds. One diarist involved in a community response, for example, described donations of food from local restaurants, meals being prepared in a school kitchen and resources being gathered and packaged in space made available by a community hub (Diarist11-Week1). Others referred to restaurants and cafés delivering to vulnerable people (Diarist10-Week1) and community hampers for a local nursing home (Diarist14-Week1).

Actors, activities, and strategies: delivering the community response

Strategies are the means through which capacity is built or engaged (dimension 3). Chaskin suggests these often include a combination of community organising, organisational and leadership development or through fostering collective relationships amongst organisations. Strategies may operate through informal social processes, organised community processes or formal targeted efforts and, to convert these strategies into action, community capacity is engaged through agency stemming from individuals, networks, and organisations (dimension 4). The 'intent' of the community capacity building is described as the function (dimension 5).

Individual acts

Although there was a degree of strategic development, the most frequently reported approaches to community capacity building operated through informal interactions with individual residents proactively applying knowledge, skills, and resources. Ten diarists described the instrumental and emotional support they provided (or observed others providing), often to those who were elderly or shielding. Throughout the eight-week study, there were frequent descriptions of *'people helping each other out'* (Diarist1-Week3-call). Activities or functions included practical tasks (e.g., shopping, fetching prescriptions and delivery medicines) and providing support through socially distanced walks and telephone calls.

Informal networks

Community capacity was also developed through networks of relationships with neighbourhood groups and organisations acting together. Several diarists described their membership of social media groups which provided a forum for requests and offers of help from one resident to another. One diarist described how they identified a need for this and set up a street-level WhatsApp group, saying they were *'enjoying being able to support my street through social media. There is always something to offer from shopping to advice'* (Diarist 14-Week1). Another diarist was heavily involved in a response co-ordinated by a town council. This approach was enabled by local government funding and individuals who were already active in their community mobilised social enterprises, businesses, and other local

For peer review only - http://bmjopen.bmj.com/site/about/guidelines.xhtml

service providers to provide essential shopping and meals to residents at risk. Their local knowledge, ability to act quickly and being known in the community were seen to be essential components underlying their rapid community response (Diarist11-Week1-call).

The contribution of organisations

At an organisational level, Chaskin suggested that the development of community capacity *"might be reflected in the ability of such organizations to carry out their functions responsively, effectively, and efficiently as part of the larger system of actors and processes to which they are connected, within and beyond the community"* (18 p.298). Positive reporting of organisational community capacity building in the diaries largely focused on organisations adapting support service to be delivered online and there was enthusiasm when, a few weeks after the commencement of lockdown, diarists were able to engage in volunteering roles. This included one diarist who became very actively involved in an initiative to support people with mental health problems and a befriending project. There was also sympathy expressed for the response of professionals such as general practitioners and the clergy who were seen to be *'reaching out a much as they can but they can't fill the gaps'* (Diarist4-Week8).

But during the initial stages of lockdown, several diarists expressed the view that the needs of vulnerable residents were most frequently being met by individuals or community networks. Diarists expressed concern that organisations needed to find alternative ways to provide for service users reliant on them for social contact and support (e.g., Diarist1-Week2-call) and several suggested that organisations had often been slow to respond to the needs of the community or provide guidance to inform community-based action. Frustration was expressed about what was seen as the lack of effective response from *'well paid people'*: one diarist described this as *'a disconnect between 'grassroots' organisations and individuals doing stuff, and… councils and others talking about the doing'* (Diarist11-Week1-call) and another wrote about a lack of co-ordination when they did react:

> *"…there are numerous voluntary organisations that do not like working with each other, they seem to have lost focus as to why they exist. They all share one thing in common, they have definitely forgotten that they exist to serve and support the community and if an individual organisation can't provide that support, then signpost them to one which can"* (Diarist10-Week2).

It was also suggested that in some cases, organisations were unable to utilise potential community action because of pre-existing organisational arrangements, thus inhibiting the potential impact of more informal groups. One diarist reported that statutory funding was not available to community groups who were ready to respond to local need because they were not formally constituted. This was at a time when some organisations which could have been part of the community network had furloughed staff, so restricting the availability of people in key roles.

Outcomes of the increased community capacity

The outcome of the community activity (dimension 6) as identified and described by the diarists included an increase in community capacity (dimension 2) as well as outcomes relating to the tasks undertaken.

Maintaining wellbeing

For peer review only - http://bmjopen.bmj.com/site/about/guidelines.xhtml

Maintaining the wellbeing of residents at risk was highlighted as a motivation for the community responses. This support was clearly valued as one diarist wrote:

> *"People are mentioning that they will really miss us tomorrow!!! It's a very humbling feeling, knowing that a small meal, a chat and a smile can mean so much to someone…"* (Diarist11-Week3)

Several diarists also described the importance to their personal wellbeing of being engaged in community or volunteering work at such a challenging time. They described these activities as *'giving a sense of purpose'* (Diary3 wk2) and enhancing feeling of self-worth through being *'useful' 'of value'* and *'productive'* (Diarist11-Week1). Another diarist described the mutual benefit of being involved in a new support initiative:

> *"Had my first Check in and chat call today, went brilliantly on the phone for an hour and I think we both got something out of the experience. Look forward to my next chat next week"* (Diarist1-Week3).

But this activity also had personal physical and mental consequences. One diarist was heavily involved in the mobilisation of community network which collected, processed, and delivered food to vulnerable residents described how demanding it could be:

> "*Over at* [locality2] *primary school to collect pasta/soups to package and distribute later today. 11.00am, 120 food/sandwiches packages ready to load onto van, the list is growing, some residents ask to be removed as they have adequate support networks, but for every 1 we take off, 4 more ask for support. Finished delivering food around 5.45pm, really tired*" (Diarist11-Week2).

In addition to being active and engaged in proving support to others, diarists were likewise experiencing the pandemic and were dealing with the psychological impact of this. This diarist also noted the additional strain associated with being out in the community and having contact with vulnerable people:

> *"Just been told that today, will be our final day of delivering support as part of the* [organisation] *COVID-19 community response. Such a mixture of feelings. Relieved, and happy that I (along with 10 of my friends and colleagues) have so far survived contracting the virus, despite placing ourselves (and our loved ones) in danger. The stress of being in close proximity with vulnerable residents, many of them elderly, with multiple and complex health conditions has been enormous at times"* (Diary11-Week7).

New connections and information sharing

The overall perception from the diarists was that the increased contact between residents had resulted in the development of new networks and channels of communication. In turn this had led to a greater awareness of the needs of individuals within the community, information sharing and pathways for individuals and businesses to '*help out more*'. Diarists also described, through their own volunteering experiences, the new initiatives and ways of working developed by the organisations they were connected with to meet the support needs of their service users.

Longevity of the community capacity

For peer review only - http://bmjopen.bmj.com/site/about/guidelines.xhtml

The nature of the Diary Study, in gathering the perceptions of 15 individuals during the early weeks of the lockdown, meant it was not possible to comment on the extent to which the community capacity built or other outcomes were sustained beyond the diary period. Even during the period covered by the Diary Study, activities were changing and in one instance, a community response that had regularly delivered shopping and meals to more than 100 people was being '*wound down'*. In part this was an apparent reaction to fears that they were *'creating dependency'* but also with the understanding that furloughed staff were returning to work and there was some return to normality.

But several diarists, as illustrated below, did express their hope that the greater sense of community and capacity built would be sustained:

> *"Making sure we don't lose the positives There have been so many positive things that have come from the Covid situation. So much connectivity between people, huge numbers offering help, old friendships renewed, new friends being made, lots of creative solutions being identified to new and difficult problems. I really hope a lot of these really beneficial things can be captured and encouraged to still remain in place when life gets a bit more back to normal"* (Diarist4-Week8).

There was a hope that this enthusiasm could be captured but clearly also some doubt – with reference to the community connectedness they had observed, this diarist said '*What a shame we can't bottle it!'* (Diary4-Week3).

CONCLUSIONS

The research reported here aimed to explore community responses to the COVID-19 pandemic. Whilst the data from this Diary Study focused on emerging community responses rather than planned initiatives, Chaskin's exploration of community capacity proved to be a useful framework to understand the observations and experiences of the diarists during the early weeks of the first UK COVID-19 lockdown. Though actions were largely reactive, the findings did indicate that capacity building took place: participants described a sense of community, and a desire to be actively engaged; the generation of ideas and solutions to local issues as they were identified and harnessing of resources were apparent in data from diary entries and weekly calls.

Using Chaskin's framework also highlights the importance of conditioning influences and in the predicament created by the COVID-19 lockdown, the accounts of the diarists elucidating the critical role of context in the development of community capacity. Inhibiting influences include the lockdown rules, restrictions on people who were shielding and the absence of existing services and other sources of support. The diarists described how community capacity building was supported by people's physical proximity to home, the use of information technology, greater availability, and by goodwill which frequently appeared to engender a feeling of being 'in it together'.

The sample included in this study was predominantly female. The social and economic effects of the pandemic are likely to have been experienced differently for men and women. Research suggests that the burden of unpaid care work already disproportionately carried out by women was exacerbated by the pandemic in ways that have the potential to be detrimental to their wellbeing (18, 19). However, data from this study suggests that pre-pandemic volunteering experience and connections supported the participation of both male and female diarists in community responses during the pandemic. The extensive involvement reported by

For peer review only - http://bmjopen.bmj.com/site/about/guidelines.xhtml

male diarists in community capacity building activities contrasts with other research which highlights the disproportionate contribution of women as voluntary care givers during the pandemic (19). The findings presented may therefore be representative of people who were already familiar with carrying out unpaid caring and supporting roles.

The picture of the community response presented by the diaries contains insights into different population characteristics that could inform the recovery. It supports research which suggests that residents must be engaged if more systemic recovery support is to be valued e.g.(20) and reveals valuable information from members of the community about the knowledge and processes that resulted in the provision of essential support whilst there were gaps in service delivery. As the National Institute for Health and Care Excellence has indicated, the recovery period provides an opportunity for local government and health and care services to build on the strengths of communities to ensure initiatives are ‘relevant and meaningful’(21).

The main functions of the community response described by the diarists was to provide essential resources to residents at risk, and to a lesser extent, social support. The sharing of intelligence was also required to enable this to happen, a finding in line with other work which described the *‘creative solutions’* developed by residents who understand their community(22) Whilst diarists wrote about the communication of lockdown rules and government health messages, there was no evidence that this formed any part of their activities, contrasting with the type of activities described in previous epidemics(7). This finding supports the UK Government’s research that found informal volunteers were most likely to be employed maintaining social contact with less mobile neighbours and carrying out practical tasks such as shopping and collecting medicine(12).

Support was provided through community organising, primarily via individual agency and local connections and, to a lesser extent, organised community processes. This resonates with other research findings(5) where the main actors in community pandemic responses were community leaders, community and faith-based organisations, community networks, committees, and individuals. Some diarists referred to targeted efforts of larger organisations but in general the activities described were not part of a strategic approach, rather they were informal and evolving in response to the initial crisis and rapidly changing circumstances. The prominence of these individual and self-organised street-level activities appeared to be effective for those residents who were identified: practical support was provided at a time when formal support services were not available or not able to cope. Further supporting the findings of Gilmore et al.(5) there was no discussion in the diaries about community involvement in wider strategic decision-making.

The greater community capacity generated during the time covered by the Diary Study appears to have been created through a wider awareness of need within the community and the development of channels to share this information and respond to need (e.g. through social media groups). Other outcomes include access to new sources of support such as co-walking and befriending which enabled the wellbeing of residents at risk to be maintained. It is also important to acknowledge the personal toll for those involved in a community response: volunteers were similarly experiencing the lockdown with its associated strain on themselves, their families, and acquaintances in addition to experiencing anxieties about their contact with infected and clinically vulnerable people. This raises questions about appropriate support and the resources required to sustain successful elements of community-based responses during the COVID-19 recovery and beyond.

For peer review only - http://bmjopen.bmj.com/site/about/guidelines.xhtml

In contrast with other community action frameworks, the concepts of empowerment and control do not feature prominently in Chaskin's dimensions of community capacity building. Similarly, the processes and outcomes described by the diarists did not include references to feelings of empowerment and control. One apparent reason for this may be that the unique context generated by the COVID-19 lockdown meant that the singular concern of many individuals, networks and organisations was to meet the immediate needs of residents most at risk. Consequently, in this context, Chaskin's framework was useful to describe this *'inward gaze'*(13) with its focus on, for example, community capacities, and neighbourhood conditions. But moving forward, and beyond the COVID-19 recovery, it is argued that power and control need to be integral elements in the consideration of community capacity, requiring an *'outward gaze'* on the political and social change required to enable structural gaps and inequalities to be addressed.

The risk of community capacity building being considered out with empowerment and control is a particular concern at a time when there are reductions in state spending and, as Popay et al.(13) argue, pressure to focus on *'equipping communities to use their 'assets' to manage 'shocks' like COVID-19—to adapt to, rather than transform, existing inequalities'* (p.8). With reduced public expenditure, process of 'citizen-shift' has also been described whereby interventions are increasingly individualised and service providers are enabled to create upstream energy from grassroots level to mobilise against inequalities(23). This is an important area for future research and could inform strategies to "build back better".

Some potential limitations of the study should be noted. Firstly, the small participant sample (n=15), largely reflected an older and predominantly female demographic and people, as Public Advisers, who were already connected into community and volunteering networks. Although not necessarily a limitation, the findings may therefore not be reflective of the general population. Second, although the findings of this study suggest that community capacity building took place, the short-term nature of the study means it is not possible to assess the extent to which it was sustained or whether there was a lasting impact. Finally, the diary entries and calls also do not provide a complete picture of activity with local communities, rather they are the observations and experiences these individuals chose to report: the method adopted meant the agenda was set by diarists and researchers did not ask diarists about any possible 'gaps' in their accounts.

There have been few qualitative diary studies completed during crisis situations(5,10,11,12) and the findings of this research suggest that the combination of written diaries and weekly calls was a useful way to gather the experiences and observations of participants during the COVID-19 lockdown. The study also contributes to building wider knowledge around community responses in the COVID-19 pandemic.

The findings illuminate the community responses experienced and observed at a time of considerable uncertainty and threats to wellbeing resulting from the COVID-19 pandemic. Chaskin's community capacity building framework focused analytical attention on the conditional influences and underlying characteristics of community capacity in addition to the strategies, actors, intent, and outcomes. This demonstrated the complexity of the situation and the central importance of the specific context and of individual agency: residents were engaged (sometimes intensively) in developing links, solutions, working with what they had and, in some cases, with organisations that were in a position to respond. This confirms the likelihood that many communities could be left behind if there were not equipped individuals

For peer review only - http://bmjopen.bmj.com/site/about/guidelines.xhtml

and/or community networks able fill the gaps in the organisational response, at least in the short-term. This could lead to increased health inequalities.

A number of directions for future research have emerged from the study. These include insights from residents who began their volunteering journey during the pandemic and looking at the impact of the community response on the wellbeing of those involved as well as those being supported. Investigating whether individuals and community networks involved in the immediate response were mobilised in the recovery post pandemic would be valuable: embedding an *'outward gaze'* into future research could also ascertain the extent to which community empowerment and involvement in wider decision-making results in strategic dimension to community capacity building post COVID-19(5). Identifying commonalities or differences in experiences across communities could also provide valuable information about inhibiting influences for 'communities left behind. In addition, this could also help us to explore relationships and networks that have been the most powerful in responding to local needs.

FUNDING
This research was funded by the National Institute for Health Research (NIHR) Applied Research Collaboration North West Coast (ARC-NWC). The views expressed are those of the author(s) and not necessarily those of the NHS, the NIHR or the Department of Health and Social Care.

ETHICS APPROVAL
Ethics approval was granted by Lancaster University Faculty of Health and Medicine's Ethics Committee in April 2020 (FHMREC19076).

CONFLICT OF INTERESTS
The author(s) declared no potential conflicts of interest with respect to the research, authorship and/or publication of this article.

AUTHOR CONTRIBUTION
Fiona Ward designed the study, developed the protocol, co-ordinated the data collection, analysed the data, wrote the manuscript; Emma Halliday designed the study, collected and analysed the data, reviewed the manuscript. Vivien Holt collected the data and reviewed the manuscript; Koser Khan collected the data and reviewed the manuscript; Gill Sadler collected the data; Paula Wheeler collected the data and reviewed the manuscript; Joanna Goldthorpe wrote the manuscript and provided methodological and analytical support.

The authors wish to thank all ARC-NWC Public Advisers who participated in the study and to Alan Price and Neil Joseph who reviewed the paper. Thanks also to Olga Angulo-Judez, Lancaster University, who provided technical support and training for diarists in the use of online methods.
This paper is dedicated to the memory of Gill Sadler who sadly passed away in January 2021.

DATA AVIAILABILITY STATEMENT
The data underlying this article cannot be shared publicly due to the need to protect the privacy and confidentiality of individuals that participated in the study. The data will be shared on reasonable request to the corresponding author.

REFERENCES

For peer review only - http://bmjopen.bmj.com/site/about/guidelines.xhtml

1. Sobande F. 'We're all in this together': Commodified notions of connection, care and community in brand responses to COVID-19. European Journal of Cultural Studies. 2020;23(6):1033-7.
2. Bambra C, Riordan R, Ford J, Matthews F. The COVID-19 pandemic and health inequalities. J Epidemiol Community Health. 2020;74(11):964-8.
3. Mishra V, Seyedzenouzi G, Almohtadi A, Chowdhury T, Khashkhusha A, Axiaq A, et al. Health inequalities during COVID-19 and their effects on morbidity and mortality. Journal of healthcare leadership. 2021;13:19.
4. Marmot M, Allen J, Goldblatt P, Herd E, Morrison J. Build back fairer: the COVID-19 Marmot review. The pandemic, socioeconomic and health inequalities in England. 2020.
5. Gilmore B, Ndejjo R, Tchetchia A, De Claro V, Mago E, Lopes C, et al. Community engagement for COVID-19 prevention and control: a rapid evidence synthesis. BMJ global health. 2020;5(10):e003188.
6. Hocevar KP, Metzger, M., & Flanagin, A. J. Source Credibility, Expertise, and Trust in Health and Risk Messaging. Oxford Research Encyclopedia of Communication: Oxford University Press; 2017.
7. Ghio D, Lawes-Wickwar S, Tang MY, Epton T, Howlett N, Jenkinson E, et al. What influences people's responses to public health messages for managing risks and preventing infectious diseases? A rapid systematic review of the evidence and recommendations. 2020.
8. Haines A, de Barros EF, Berlin A, Heymann DL, Harris MJ. National UK programme of community health workers for COVID-19 response. The Lancet. 2020;395(10231):1173-5.
9. South J, Stansfield J, Amlôt R, Weston D. Sustaining and strengthening community resilience throughout the COVID-19 pandemic and beyond. Perspectives in Public Health. 2020;140(6):305-8.
10. Mak HW, Fancourt D. Predictors of engaging in voluntary work during the Covid-19 pandemic: analyses of data from 31,890 adults in the UK. Perspectives in Public Health. 2020:1757913921994146.
11. Carlsen HB, Toubøl J, Brincker B. On solidarity and volunteering during the COVID-19 crisis in Denmark: the impact of social networks and social media groups on the distribution of support. European Societies. 2021;23(sup1):S122-S40.
12. Department of Digital C, Media & Sport & Office for Civil Society. A Look at Volunteering during the Response to COVID-19. 2021. A look at volunteering during the response to COVID-19 2021.
13. Popay J, Whitehead M, Ponsford R, Egan M, Mead R. Power, control, communities and health inequalities I: theories, concepts and analytical frameworks. Health promotion international. 2020.
14. Chaskin RJ. Building community capacity: A definitional framework and case studies from a comprehensive community initiative. Urban affairs review. 2001;36(3):291-323.
15. Medd W, Deeming H, Walker G, Whittle R, Mort M, Twigger-Ross C, et al. The flood recovery gap: a real-time study of local recovery following the floods of J une 2007 in H ull, N orth E ast E ngland. Journal of Flood Risk Management. 2015;8(4):315-28.
16. Mort M, Convery I, Baxter J, Bailey C. Psychosocial effects of the 2001 UK foot and mouth disease epidemic in a rural population: qualitative diary based study. Bmj. 2005;331(7527):1234.
17. Herron R, Dansereau L, Wrathall M, Funk L, Spencer D. Using a flexible diary method rigorously and sensitively with family carers. Qualitative health research. 2019;29(7):1004-15.
18. Andersen D, Toubøl J, Kirkegaard S, Bang Carlsen H. Imposed volunteering: Gender and caring responsibilities during the COVID-19 lockdown. The Sociological Review. 2022;70(1):39-56.
19. Women more likely than men to have tried to help others amid COVID-19 outbreak [press release]. 2020.
20. Harkins C. Supporting community recovery and resilience in response to the COVID-19 pandemic–a rapid review of evidence. Glasgow; 2020.

For peer review only - http://bmjopen.bmj.com/site/about/guidelines.xhtml

21. Maiden H, Jagroo J, Shearn P, Wasielewska A, Nolan K, Baillie N. Spotlight on community engagement: NICE resources in the context of COVID-19—NICE public health guidance update. Journal of Public Health (Oxford, England). 2021.

22. McMillan R. Learning from nine months of crisis response: Local Trust; 2020 [Available from: https://localtrust.org.uk/news-and-stories/blog/learning-from-nine-months-of-crisis-response/

23. Williams O, Fullagar S. Lifestyle drift and the phenomenon of 'citizen shift'in contemporary UK health policy. Sociology of health & illness. 2019;41(1):20-35.

For peer review only

For peer review only - http://bmjopen.bmj.com/site/about/guidelines.xhtml

Figure 1. Chaskin framework dimensions applied to Diary Project findings

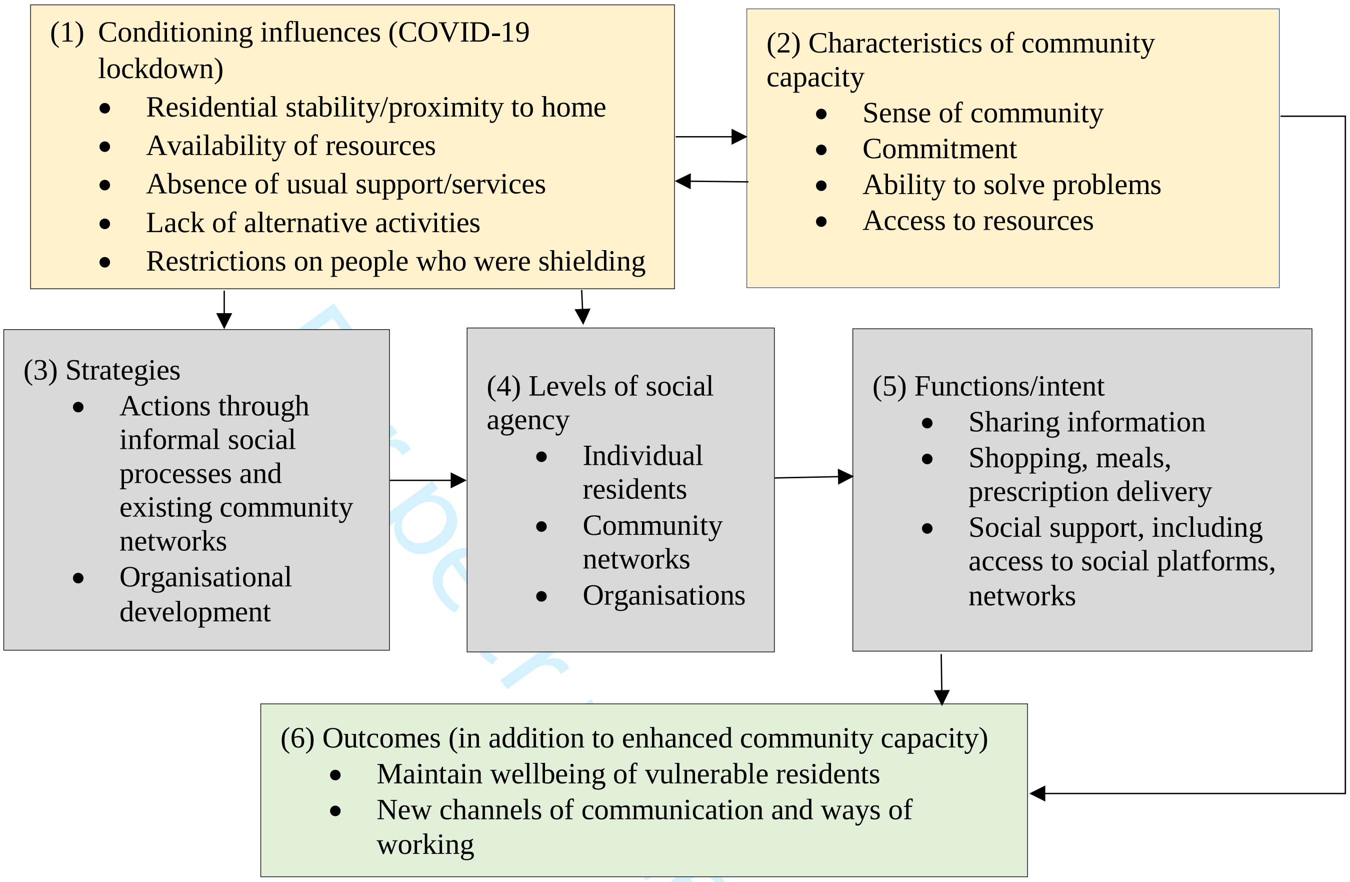

For peer review only

COREQ (COnsolidated criteria for REporting Qualitative research) Checklist

A checklist of items that should be included in reports of qualitative research. You must report the page number in your manuscript where you consider each of the items listed in this checklist. If you have not included this information, either revise your manuscript accordingly before submitting or note N/A.

Topic	Item No.	Guide Questions/Description	Reported on Page No.
**Domain 1: Research team and reflexivity**			
Personal characteristics			
Interviewer/facilitator	1	Which author/s conducted the interview or focus group?	
Credentials	2	What were the researcher's credentials? E.g. PhD, MD	
Occupation	3	What was their occupation at the time of the study?	
Gender	4	Was the researcher male or female?	
Experience and training	5	What experience or training did the researcher have?	
Relationship with participants			
Relationship established	6	Was a relationship established prior to study commencement?	
Participant knowledge of the interviewer	7	What did the participants know about the researcher? e.g. personal goals, reasons for doing the research	
Interviewer characteristics	8	What characteristics were reported about the inter viewer/facilitator? e.g. Bias, assumptions, reasons and interests in the research topic	
**Domain 2: Study design**			
Theoretical framework			
Methodological orientation and Theory	9	What methodological orientation was stated to underpin the study? e.g. grounded theory, discourse analysis, ethnography, phenomenology, content analysis	
Participant selection			
Sampling	10	How were participants selected? e.g. purposive, convenience, consecutive, snowball	
Method of approach	11	How were participants approached? e.g. face-to-face, telephone, mail, email	
Sample size	12	How many participants were in the study?	
Non-participation	13	How many people refused to participate or dropped out? Reasons?	
Setting			
Setting of data collection	14	Where was the data collected? e.g. home, clinic, workplace	
Presence of non-participants	15	Was anyone else present besides the participants and researchers?	
Description of sample	16	What are the important characteristics of the sample? e.g. demographic data, date	
Data collection			
Interview guide	17	Were questions, prompts, guides provided by the authors? Was it pilot tested?	
Repeat interviews	18	Were repeat inter views carried out? If yes, how many?	
Audio/visual recording	19	Did the research use audio or visual recording to collect the data?	
Field notes	20	Were field notes made during and/or after the inter view or focus group?	
Duration	21	What was the duration of the inter views or focus group?	
Data saturation	22	Was data saturation discussed?	
Transcripts returned	23	Were transcripts returned to participants for comment and/or	

For peer review only

Topic	Item No.	Guide Questions/Description	Reported on Page No.
		correction?	
**Domain 3: analysis and findings**			
Data analysis			
Number of data coders	24	How many data coders coded the data?	
Description of the coding tree	25	Did authors provide a description of the coding tree?	
Derivation of themes	26	Were themes identified in advance or derived from the data?	
Software	27	What software, if applicable, was used to manage the data?	
Participant checking	28	Did participants provide feedback on the findings?	
Reporting			
Quotations presented	29	Were participant quotations presented to illustrate the themes/findings? Was each quotation identified? e.g. participant number	
Data and findings consistent	30	Was there consistency between the data presented and the findings?	
Clarity of major themes	31	Were major themes clearly presented in the findings?	
Clarity of minor themes	32	Is there a description of diverse cases or discussion of minor themes?	

Developed from: Tong A, Sainsbury P, Craig J. Consolidated criteria for reporting qualitative research (COREQ): a 32-item checklist for interviews and focus groups. *International Journal for Quality in Health Care*. 2007. Volume 19, Number 6: pp. 349 – 357

Once you have completed this checklist, please save a copy and upload it as part of your submission. DO NOT include this checklist as part of the main manuscript document. It must be uploaded as a separate file.

For peer review only
